# Lysophosphatidylcholine Promotes Phagosome Maturation and Regulates Inflammatory Mediator Production Through the Protein Kinase A–Phosphatidylinositol 3 Kinase–p38 Mitogen-Activated Protein Kinase Signaling Pathway During *Mycobacterium tuberculosis* Infection in Mouse Macrophages

**DOI:** 10.3389/fimmu.2018.00920

**Published:** 2018-04-27

**Authors:** Hyo-Ji Lee, Hyun-Jeong Ko, Dong-Kun Song, Yu-Jin Jung

**Affiliations:** ^1^Department of Biological Sciences, Kangwon National University, Chuncheon, South Korea; ^2^Institute of Life Sciences, Kangwon National University, Chuncheon, South Korea; ^3^College of Pharmacy, Kangwon National University, Chuncheon, South Korea; ^4^Department of Pharmacology, College of Medicine, Hallym University, Chuncheon, South Korea

**Keywords:** *Mycobacterium tuberculosis*, macrophage, lysophosphatidylcholine, phagosome maturation, inflammation

## Abstract

Tuberculosis is caused by the infectious agent *Mycobacterium tuberculosis* (Mtb). Mtb has various survival strategies, including blockade of phagosome maturation and inhibition of antigen presentation. Lysophosphatidylcholine (LPC) is a major phospholipid component of oxidized low-density lipoprotein and is involved in various cellular responses, such as activation of second messengers and bactericidal activity in neutrophils. In this study, macrophages were infected with a low infectious dose of Mtb and treated with LPC to investigate the bactericidal activity of LPC against Mtb. In macrophages infected with Mtb strain, H37Ra or H37Rv, LPC suppressed bacterial growth; however, this effect was suppressed in bone marrow-derived macrophages (BMDMs) isolated from G2A (a G protein-coupled receptor involved in some LPC actions) knockout mice. LPC also promoted phagosome maturation *via* phosphatidylinositol 3 kinase (PI3K)–p38 mitogen-activated protein kinase (MAPK)-mediated reactive oxygen species production and intracellular Ca^2+^ release during Mtb infection. In addition, LPC induced increased levels of intracellular cyclic adenosine monophosphate (cAMP) and phosphorylated glycogen synthase kinase 3 beta (GSK3β) in Mtb-infected macrophages. Protein kinase A (PKA)-induced phosphorylation of GSK3β suppressed activation of NF-κB in LPC-treated macrophages during Mtb infection, leading to decreased secretion of pro-inflammatory cytokines and increased secretion of anti-inflammatory cytokines. These results suggest that LPC can effectively control Mtb growth by promoting phagosome maturation *via* cAMP-induced activation of the PKA–PI3K–p38 MAPK pathway. Moreover, LPC can regulate excessive production of pro-inflammatory cytokines associated with bacterial infection of macrophages.

## Introduction

Tuberculosis (TB) is the most important bacterial infection and remains a major cause of morbidity and mortality worldwide. According to World Health Organization reports, an estimated one-third of the world’s population is infected with *Mycobacterium tuberculosis* (Mtb) in an asymptomatic state, which is defined as having latent TB (1, 2). After Mtb internalization, monocyte-derived macrophages, dendritic cells (DCs), and neutrophils participate in the phagocytic process and express inflammatory mediators, including pro- and anti-inflammatory cytokines, chemokines, and inducible nitric oxide synthase (3, 4). However, Mtb has several mechanisms to evade host immune responses, such as phagosome–lysosome fusion interference, inhibition of phagosome acidification, inflammatory immune suppression, and host cell death modulation (5). Live Mtb induces low levels of cytosolic Ca^2+^ release, which is correlated with inhibition of phagosome–lysosome fusion, suggesting that Ca^2+^-induced intracellular signaling pathways contribute to the intracellular pathogen survival and pathogenesis of TB (6, 7). Other immune evasion mechanisms include secretion of enzymes by Mtb, such as superoxide dismutase or catalases, which are antagonistic to reactive oxygen intermediates, or inhibition of macrophage apoptosis (8).

Phagocytosis is an essential innate immune defense mechanism for innate immune cells to eliminate microbes or apoptotic cells (9). After a particle is internalized, phagosomes undergo several “maturation” stages through a series of increasingly acidified membrane-bound structures (10). Many of the fusion and fission events in phagosome maturation are mediated by phosphatidylinositol 3 kinase (PI3K), which generates phosphatidylinositol 3-phosphate from phosphatidylinositol 3. PI3K is involved in anchoring early endosomal antigen 1 (EEA1) and other proteins to the phagosomal membrane (11). Ca^2+^ is required for the process of phagosome maturation by surrounding nascent phagosomes (7). Ca^2+^ promotes the fusion of the phagosome to granules containing lytic enzymes and the assembly and activation of the reactive oxygen species (ROS)-generating nicotinamide adenine dinucleotide phosphate oxidase complex (12, 13). Therefore, determining the underlying mechanism of modulation of phagosome maturation is more important to control invading pathogens, such as Mtb.

Lysophosphatidylcholine (LPC) is a major component of oxidized low-density lipoprotein and is a natural agonist of the G protein-coupled receptor G2A (14), which modulates T cell migration, macrophage, and neutrophil activation and phagocytic clearance of apoptotic cells (15–18). These LPC-dependent effects of G2A contribute to the mechanisms controlling initiation and resolution of inflammation in response to infection and acute or chronic inflammatory diseases, such as sepsis or autoimmune disease, respectively (19). LPC can protect mice from cecal ligation and puncture (CLP, a clinically reliable model of sepsis)-induced lethality (20). In addition, LPC has direct antibacterial activities in Methicillin-resistant *Staphylococcus aureus via* inducing membrane depolarization of the bacteria (21), or enhances the antimicrobial ability of neutrophils to remove ingested bacteria (20, 22). Apart from these findings, LPC has not been proven to control *Leishmania major*, even though it enhanced the ability of phagocytosis in DCs (23). Brancucci et al. showed that host-derived LPC controls sexual stage differentiation of *Plasmodium falciparum*, which is a causative agent of malaria (24). Therefore, LPC has a variety functions relevant to the infections. However, the role of LPC in innate immune regulation in macrophages during Mtb infection has not been elucidated, although LPC has a protective effect against sepsis-induced lethality and antimicrobial activity in neutrophils and DCs. In this report, to identify the immunomodulatory activity of LPC in Mtb-infected mouse macrophages, we infected macrophages with a low infectious dose of Mtb and treated them with LPC. Our findings showed that LPC inhibited intracellular bacterial growth by promoting phagosome maturation *via* an increase in cytosolic Ca^2+^ release, which was induced through G2A-mediated activation of phospholipase C (PLC) during Mtb infection. Secretion of ROS and nitric oxide (NO) was enhanced through cyclic adenosine monophosphate (cAMP)-induced activation of protein kinase A (PKA) in LPC-treated cells during Mtb infection. Furthermore, production of pro-inflammatory cytokines was downregulated by increased phosphorylation of glycogen synthase kinase 3 beta (GSK3β) (Ser 9) through PKA-mediated signaling in Mtb-infected macrophages. These results suggest that LPC can effectively control Mtb growth by promoting phagosome maturation *via* cAMP-induced activation of the PKA–PI3K–p38 mitogen-activated protein kinase (MAPK) pathway, which may avoid excessive inflammation associated with macrophage infection. These findings indicate that LPC treatment might be beneficial for patients with TB.

## Materials and Methods

### Ethics Statement

This study was managed in strict accordance with the Guide for the Care and Use of Laboratory Animals (25), and all experimental animals procedures used in this study were handled using a protocol approved by the Institutional Animal Care and Use Committee of Kangwon National University (KIACUC, KW-130613-1).

### Cell Culture

The mouse macrophage cell line, Raw264.7, was purchased from ATCC (ATCC, Rockville, MD, USA) and maintained in RPMI-1640 (Cellgro, Herndon, VA, USA) culture medium containing 10% fetal bovine serum (FBS; Atlas Biologicals, Fort Collins, CO, USA) and penicillin/streptomycin (Corning Incorporated, Corning, NY, USA) at 37°C with 5% CO_2_.

### Cell Treatments

Raw264.7 cells were pretreated with the following kinase inhibitors for 1 h: H-89 (PKA: 10 µM), SB203580 (p38: 10 µM), wortmannin (100 nM), and SB216763 (GSK3β: 10 µM). To verify the role of ROS, Raw264.7 cells were pretreated with diphenyleneiodonium (DPI) (10 µM) or apocynin (10 µM) for 1 h. To inhibit vacuolar H^+^-ATPase, cells were pretreated with bafilomycin A1 (1 µM) for 2 h. To remove intracellular Ca^2+^, Raw264.7 cells were pretreated with 1,2-bis(2-aminophenoxy)ethane-*N*,*N*,*N*′,*N*′-tetraacetic acid tetrakis/acetoxymethyl ester (BAPTA/AM) (30 µM) for 30 min. To inhibit the activation of PLC, cells were pretreated with D609 (10 µM) for 12 h. To inhibit the production of NO, cells were pretreated with l-N6-(1-iminoethyl)lysine (l-NIL) (30 µM) for 1 h.

### Isolation and Culture of Mouse Bone Marrow-Derived Macrophages (BMDMs)

Bone marrow cells were isolated from femurs and tibiae from 8-week-old C57BL/6 or G2A knockout (KO) mice, and all muscle and adipose tissues were removed the isolated bones. Cells were centrifuged for 5 min at 1,000 rotations per min (RPM) and then resuspended in RPMI-1640 medium containing 10% FBS. The cells were incubated for 2 h at 37°C with 5% CO_2_, non-adherent cells were collected and centrifuged, and red blood cells were removed. The cells were grown in the presence of 30% supernatant from L929 cells for 4 days. Non-adherent cells were removed by washing with phosphate-buffered saline (PBS), and adherent cells were used the following day for infection.

### Mice

This study was managed in strict accordance with the Guide for the Care and Use of Laboratory Animals (25), and all experimental animals procedures used in this study were handled using a protocol approved by the Institutional Animal Care and Use Committee of Kangwon National University (KIACUC, KW-130613-1). Five-week-old male C57BL/6 mice purchased from Nara Bio (Nara Bio., Seoul, Korea) were used in this study. G2A KO mice were generously provided by Dr. Janusz H. Kabarowski (University of Alabama at Birmingham). All mice were housed under specific pathogen-free conditions with food and water provided *ad libitum*. All mice were sacrificed at 8–10 weeks old to isolate bone marrow-derived cells.

### Reagents

H-89 was purchased from Invivogen (San Diego, CA, USA). SB203580 and wortmannin were purchased from Cell Signaling Technology (Danvers, MA, USA). SB216763 was purchased from Tocris Bioscience (Ellisville, MO, USA). Other reagent sources were as follows: bafilomycin A1 (LC Laboratories, Woburn, MA, USA), DPI (Calbiochem, San Diego, CA, USA), apocynin (Sigma-Aldrich, St. Louis, MO, USA), l-NIL (Calbiochem), 2′,7′-dichlorofluorescein diacetate (DCFH-DA) (Calbiochem), DND-160 (Molecular Probes, Eugene, OR, USA), Fluo-4/AM (Molecular Probes, USA), D609 (Sigma-Aldrich, St. Louis, MO, USA), and BAPTA/AM (Sigma-Aldrich, St. Louis, MO, USA).

### Bacterial Strains and Culture Conditions

*Mycobacterium tuberculosis* H37Rv and H37Ra strains were used as virulent and attenuated strains, respectively. Mtb H37Rv and H37Ra were generously provided by Sang-Nae Cho (Yonsei University). Bacterial cultures were performed as previously described (26). Briefly, Mtb was grown at 37°C in Middlebrook 7H9 broth (Difco Laboratories, Detroit, MI, USA) supplemented with 10% ADC (5% bovine albumin, 2% dextrose, 0.03% catalase, and 0.85% sodium chloride) containing 0.2% glycerol for 3 weeks. After 3 weeks, Mtb was harvested, aliquoted, and maintained at −70°C until used.

### Determination of Colony-Forming Units (CFUs)

Raw264.7 cells and BMDMs were infected with Mtb strain H37Rv and H37Ra for 4 h and then washed with PBS twice to remove extracellular bacteria. Infected macrophages were lysed with 0.1% saponin for 10 min at 37°C with 5% CO_2_ and then serially diluted in PBS. After dilution of the bacteria, 50 µl of three dilutions was plated on Middlebrook 7H10 agar with 10% OADC (0.06% oleic acid, 5% bovine albumin, 2% dextrose, 0.03% catalase, and 0.85% sodium chloride) in triplicate and incubated at 37°C. CFUs were counted after 21 days of incubation.

### Enzyme-Linked Immunosorbent Assay (ELISA)

Enzyme-linked immunosorbent assay was performed with suspensions of macrophages infected for the indicated durations according to the manufacturer’s protocols. The plate was read at an absorbance of 405 nm using a microplate reader (BioTek Instruments Inc., Winooski, VT, USA). Recombinant murine TNF-α, IL-6, or IL-10 was used as a standard.

### NO Assay

Nitric oxide production was measured as the total nitrite concentration using a NO detection kit (Intron Biotechnology Inc., Kyungki-Do, Korea) according to the manufacturer’s procedure. Cells were infected with H37Ra or H37Rv treated with LPC, and cell supernatants were collected at the indicated time points. NO production was assessed as previously described (26). Briefly, cell supernatants were mixed with N1 buffer (sulfanilamide in the reaction buffer) for 10 min, and N2 buffer (naphthylethylenediamine in stabilizer buffer) was added to the mixture for 10 min. The absorbance at 540 nm was measured using a microplate reader. The standard curve was measured with purified nitrite.

### Measurement of Intracellular ROS

Intracellular ROS was determined using a fluorescence-based dye, DCFH-DA. In short, cells were allowed to adhere to coverslips in 12-well plates and were infected with Mtb together with LPC treatment. The cells were treated with RPMI-1640 medium containing DCFH-DA for 20 min at 37°C with 5% CO_2_, washed twice with PBS and fixed with PBS containing 4% paraformaldehyde. Coverslips were mounted in Fluoromount-G™ (SouthernBiotech, Birmingham, AB, USA) and examined by confocal microscopy (FV1000 SPD, Olympus, Tokyo, Japan). For flow cytometry, the cells were rinsed with PBS, and the fluorescence intensity of dihydrodichlorofluorescein (DCF) was measured using a FACSCalibur (BD Biosciences, San Jose, CA, USA). The data were plotted and analyzed using CellQuest software.

### Measurement of Lysosomal pH

Phagosomal acidification was measured using LysoSensor Yellow/Blue DND-160, a fluorescent ratiometric pH indicator. Briefly, Raw264.7 cells were infected with H37Ra [multiplicity of infection (MOI) of 5], stimulated with LPC for 3 h and then labeled with 5 µM LysoSensor Yellow/Blue DND-160 for 10 min at 37°C with 5% CO_2_ in complete medium. Excess dye was then removed with PBS, and images were obtained using a confocal microscope.

### Immunofluorescent Staining

Macrophages were allowed to adhere to coverslips in 12-well plates for 24 h and were then infected with fluorescent [fluorescein isothiocyanate (FITC)]-labeled Mtb H37Ra at an MOI of 5 together with LPC treatment. After infection, the cells were washed with PBS and fixed with PBS containing 4% paraformaldehyde overnight at 4°C. Immunostaining was performed as previously described (26). Coverslips were mounted in Fluoromount-G™ and examined by confocal microscopy. The number of macrophages was counted in the microscopic field, and the ratio of fluorescent cells to total cells in the field was calculated. A total of 3–5 fields were examined for each coverslip. Data were calculated from 30 to 50 cells in acquired images per time point per experiment. The mean ± SD ratio was then calculated from all microscopic fields examined. The degree of colocalization of Mtb with each phagosome marker was expressed as the mean ± SD ratio of double-positive cells. The ratio of colocalization of each group was calculated from Mtb infection alone.

### Intracellular Ca^2+^ Imaging

Raw264.7 cells cultured on glass-bottom 18-mm dishes were loaded with 3 µM of the fluorescent indicator Fluo-4/AM in RPMI-1640 for 30 min at 37°C with 5% CO_2_. After labeling, the cells were washed two times and then stained with 4′,6-diamidino-2-phenylindole (DAPI) for 5 min. Images were obtained by confocal microscopy.

### Measurement of cAMP Release

Levels of intracellular cAMP were determined by enzyme immunoassay (EIA) using a cAMP EIA kit (Cayman, Ann Arbor, MI, USA). cAMP EIA was performed according to the manufacturer’s instructions. Briefly, Raw264.7 cells were seeded into a 60-mm culture dish and cultured in complete medium. Subsequently, the cells were infected with H37Ra stimulated with LPC for 15 min. The medium was then changed to 0.1 M HCl, and the cells were further incubated for 20 min at room temperature. The cell suspensions were collected, and the lysates were centrifuged at 1,000 RPM for 10 min. The samples were transferred into a microplate, and the cAMP levels were determined by reading at 405 nm using an automated ELISA reader. Each sample was analyzed in triplicate, and the values represent the means ± SD of triplicates.

### Western Blot Assay

Macrophages were infected with Mtb for the indicated times. Cells were lysed with lysis buffer consisting of 10 mM Tris–HCl, 1 mM EDTA, 140 mM NaCl, 0.1% DOC, 0.1% SDS, and Triton X-100 containing complete protease inhibitor cocktail (Calbiochem, San Diego, CA, USA). Western blotting was performed as previously described (27). Anti-phospho-GSK3β, anti-IκBα, anti-cathepsin D, and anti-β-actin were purchased from Santa Cruz Biotechnology (Santa Cruz, CA, USA). Anti-phospho-PI3K, anti-PI3K, anti-phospho-p38, anti-p38, anti-phospho-ERK1/2, anti-phospho-MEK1/2, anti-phospho-JNK1/2, anti-phospho-IκBα, and anti-IRAK-M were purchased from Cell Signaling Technology.

### Statistical Analysis

The data were obtained from independent experiments. For statistical analysis, data obtained from independent experiments (mean ± SD) were analyzed using two-tailed Student’s *t*-test and one-way ANOVA followed by Tukey’s *post hoc* test for multiple comparisons. Data were graphed and analyzed using GraphPad Prism software (GraphPad Software, La Jolla, CA, USA). Values of *p* < 0.05 were considered significant. Statistical significance was indicated as *p < 0.05; **p < 0.01; ***p < 0.001, and not significant (*p* > 0.05).

## Results

### LPC Controls the Growth of Mtb Without Excessive Production of Pro-Inflammatory Cytokines

**Figure 1:**
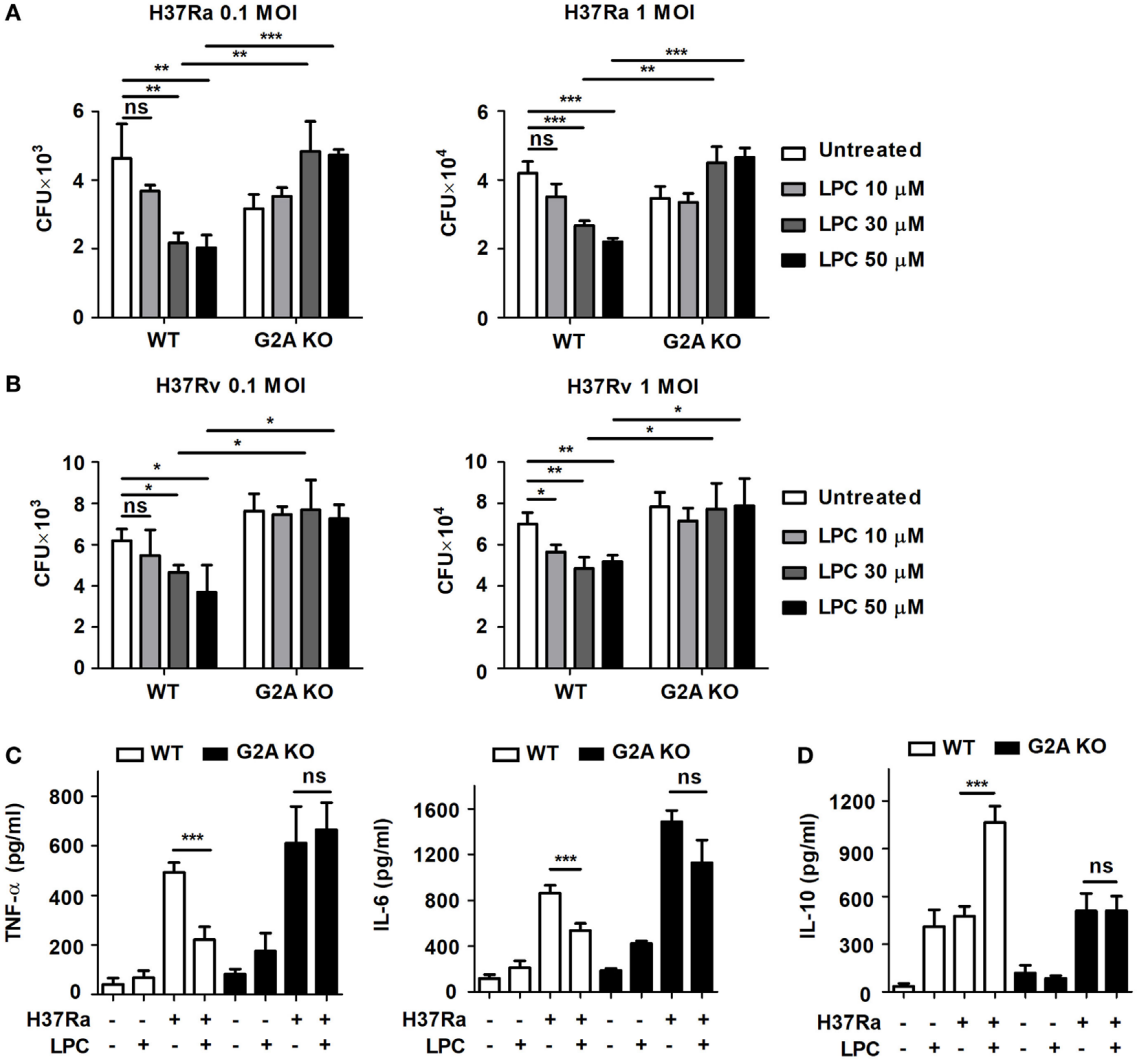
Lysophosphatidylcholine (LPC) controls *Mycobacterium tuberculosis* (Mtb) growth and inflammatory cytokine production. Bone marrow-derived macrophages (BMDMs) isolated from wild-type (WT) and G2A knockout (KO) mice were infected with avirulent Mtb H37Ra **(A)** or virulent Mtb H37Rv **(B)** [at multiplicity of infections (MOIs) of 0.1 and 1] and stimulated with different doses of LPC for 24 h. **(A,B)** Intracellular bacteria were assayed for viability based on the number of colony-forming units (CFUs) after 3 weeks. **(C,D)** Production of TNF-α and IL-6 **(C)** and IL-10 **(D)** was measured by enzyme-linked immunosorbent assay in the culture supernatant of WT or G2A KO BMDMs at 24 h. The experiments were performed in triplicate. Data obtained from independent experiments (mean ± SD) were analyzed using one-way ANOVA followed by Tukey’s *post hoc* test for multiple comparisons; *p < 0.05; **p < 0.01; ***p < 0.001, and not significant (ns) (*p* > 0.05).

Lysophosphatidylcholine can recruit immune cells and modulate their function, especially in neutrophils and T cells, and it may play a role in the control of bacterial growth during infection (28). To verify whether LPC possesses mycobactericidal activity, mouse macrophages were infected with avirulent or virulent Mtb, H37Ra or H37Rv, respectively, at an MOI of 0.1 or 1 and then treated them with LPC (Figure 1). When BMDMs isolated from wild-type (WT) C57BL/6 mice were infected with avirulent H37Ra, bacterial growth was markedly decreased in a dose-dependent manner in cells infected with both low and high doses of Mtb (Figure 1A). Moreover, treatment with LPC significantly decreased the growth of virulent H37Rv in a dose-dependent manner in BMDMs (Figure 1B). In addition, LPC decreased bacterial growth in Raw264.7 macrophages when cells were infected with avirulent H37Ra or a low dose of virulent H37Rv (Figure S1A in Supplementary Material). To further determine whether LPC could control Mtb growth *via* the G2A receptor during Mtb infection, BMDMs were isolated and differentiated from WT and G2A receptor knockout (G2A KO) mice. As shown in Figures 1A,B, LPC treatment significantly decreased bacterial growth in WT BMDMs in a dose-dependent manner; however, bacterial growth was restored in G2A KO BMDMs when cells were infected with H37Ra or H37Rv.

Next, to verify the effects of LPC on pro-inflammatory cytokine production during Mtb infection, mouse macrophages were infected with H37Ra or H37Rv and then stimulated them with LPC. Although LPC treatment increased production of the pro-inflammatory cytokines TNF-α and IL-6 in a dose-dependent manner in Mtb-infected macrophages (data not shown), the production of TNF-α and IL-6 was decreased in LPC-treated WT BMDMs after H37Ra infection (Figure 1C). However, production of the anti-inflammatory cytokine IL-10 was significantly lower in WT BMDMs infected with Mtb and treated with LPC than in cells that were either treated with LPC or infected with Mtb (Figure 1D). By contrast, production of TNF-α, IL-6, and IL-10 did not change in G2A KO BMDMs with or without LPC treatment during H37Ra infection. These results suggest that LPC can help mouse macrophages control Mtb growth without an excessive inflammatory response *via* G2A receptor engagement during infection.

### LPC Treatment Induces Activation of the PI3K–p38 MAPK Pathway in Mtb-Infected Macrophages

**Figure 2:**
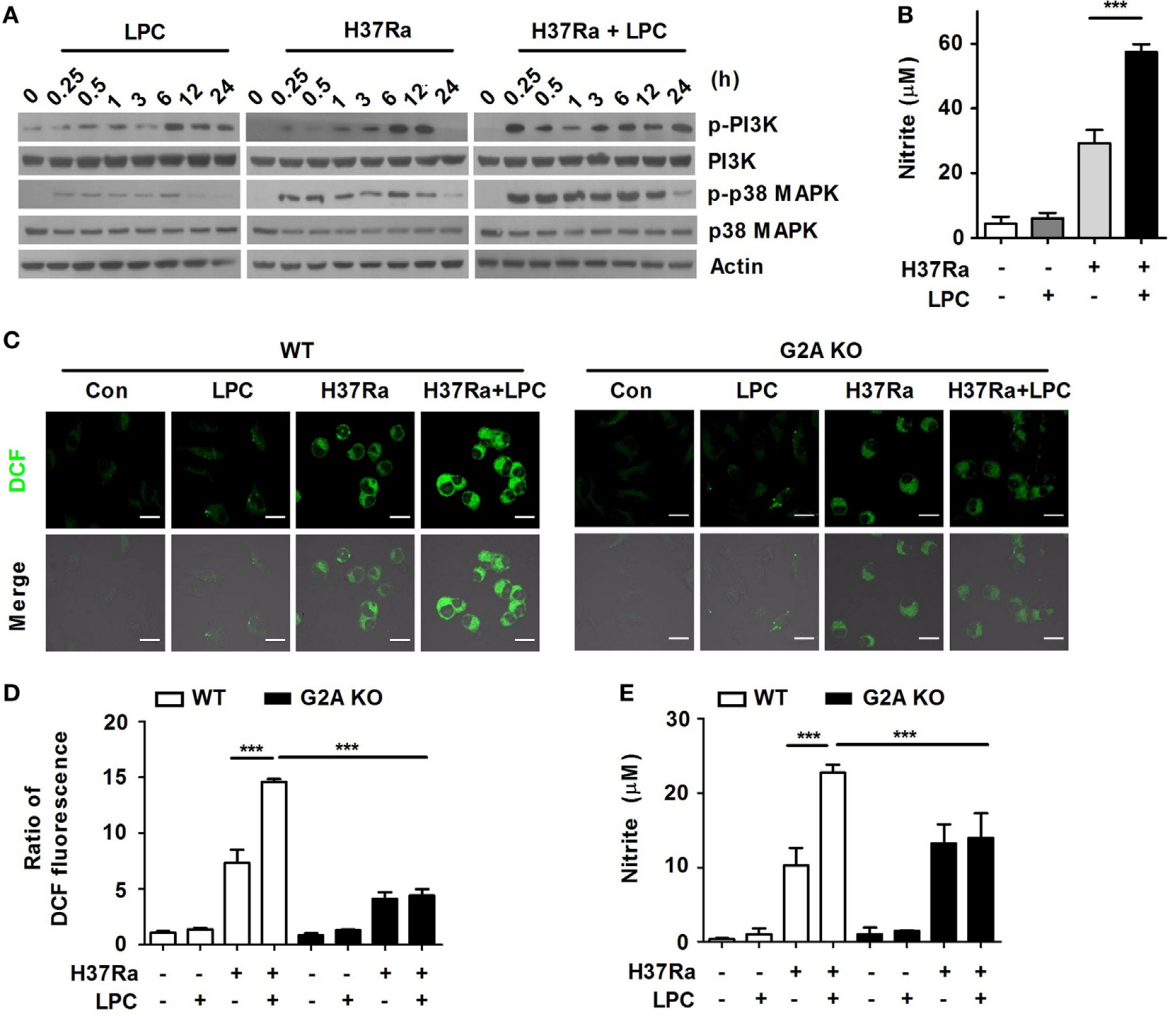
Lysophosphatidylcholine (LPC) enhances activation of phosphatidylinositol 3 kinase (PI3K)–p38 mitogen-activated protein kinase (MAPK) signaling and production of reactive oxygen species (ROS) and nitric oxide (NO) during *Mycobacterium tuberculosis* infection. **(A)** Raw264.7 cells were infected with H37Ra and treated with LPC for the indicated times. Phosphorylated and total protein levels for various MAPK signaling components were examined by Western blot analysis. **(B)** NO production was detected in cell culture supernatants at 24 h. **(C)** Intracellular ROS levels were measured based on dihydrodichlorofluorescein (DCF) fluorescence in LPC-treated wild-type (WT) and G2A knockout (KO) bone marrow-derived macrophages during H37Ra infection (multiplicity of infection of 5). **(D)** The bar graph represents the ratio of DCF fluorescence. **(E)** NO production was detected in cell culture supernatants at 24 h. The experiments were performed in triplicate (***p < 0.001).

To determine whether LPC controls bacterial growth through certain intracellular signaling mechanisms, Raw264.7 cells were infected with H37Ra and then treated them with LPC. LPC treatment rapidly enhanced the phosphorylation of PI3K and p38 MAPK after 15 min of H37Ra infection in macrophages (Figure 2A), but the phosphorylation level of MEK1/2, ERK1/2, and JNK1/2 was decreased in LPC-treated cells infected with Mtb (Figure S1C in Supplementary Material). Furthermore, LPC treatment induced a decreased level of phosphorylated IκBα and delayed degradation of IκBα in H37Ra-infected macrophages (Figure S1C in Supplementary Material).

During Mtb infection, ROS and NO production play critical roles in the control of bacterial growth (29). To verify whether LPC induces the production of radical mediators during Mtb infection, ROS and NO production was measured in supernatants from macrophages infected with H37Ra followed by LPC treatment. LPC treatment markedly increased the level of ROS (Figure S1D in Supplementary Material) as well as the production of NO in H37Ra-infected Raw264.7 cells (Figure 2B). In addition, intracellular ROS generation was significantly increased in LPC-treated WT BMDMs after Mtb infection, whereas it was not increased in G2A KO BMDMs treated with LPC during Mtb infection (Figures 2C,D). In addition, the production of NO was detected at a low level in H37Ra-infected BMDMs from G2A KO mice regardless of LPC treatment (Figure 2E). These data suggest that LPC induces the generation of ROS and NO, which mediate the PI3K–p38 signaling pathway (Figure S8 in Supplementary Material), to control Mtb growth in mouse macrophages.

### LPC Promotes the Maturation of Mtb-Containing Phagosomes to Control Bacterial Growth

Phagocytosis is an essential process in the innate immune response of professional phagocytes to kill microbial pathogens and clear apoptotic cells. Recently, the TLR signaling pathway is activated by bacteria, such as *S*. typhimurium or *E. coli*, and regulates phagocytosis at multiple steps, including internalization and phagosome maturation, through MyD88-dependent activation of p38 MAPK (30).

**Figure 3:**
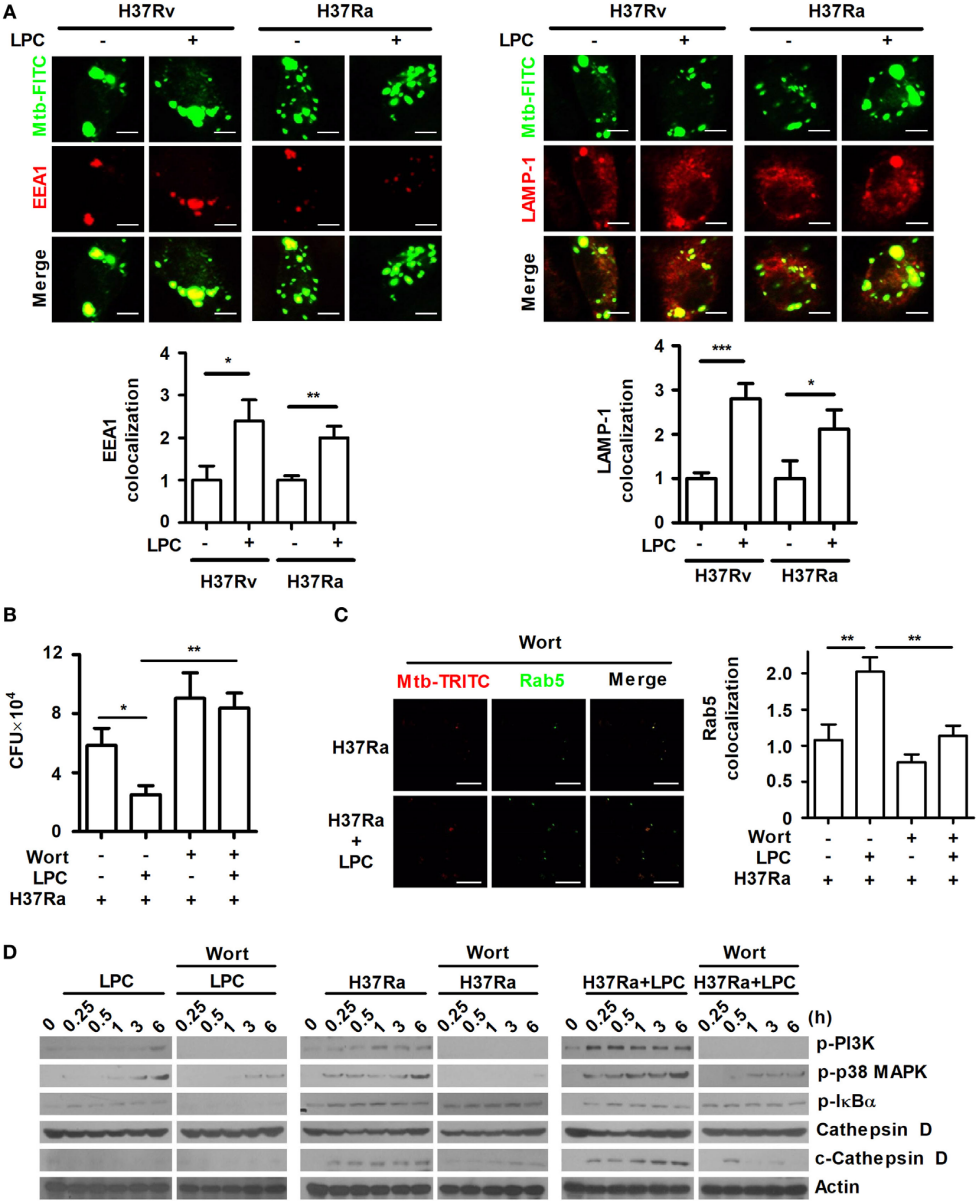
Lysophosphatidylcholine (LPC) enhances phagosome maturation *via* the phosphatidylinositol 3 kinase (PI3K)-mediated signaling pathway in *Mycobacterium tuberculosis* (Mtb)-infected macrophages. **(A)** C57BL/6 bone marrow-derived macrophages were infected with fluorescein isothiocyanate (FITC)-labeled H37Ra or H37Rv (multiplicity of infection of 5) and treated with LPC for 3 h. After infection, cells were stained with early endosomal antigen 1 (EEA1) or lysosomal-associated membrane protein 1 (LAMP-1), and Mtb colocalization with each marker was observed by confocal microscopy. The bar graphs represent the ratio of Mtb colocalization with each marker. **(B)** The growth of intracellular bacteria was determined by colony-forming units (CFUs) in H37Ra-infected Raw264.7 cells with or without pretreatment with wortmannin (Wort, 100 nM for 1 h). **(C)** After Wort pretreatment, Raw264.7 cells were infected with TRITC-labeled H37Ra and treated with LPC for 3 h. After infection, cells were stained with Ras-associated protein 5 (Rab5), and each spot was counted using a confocal microscope. The bar graph represents the ratio of Mtb colocalization with Rab5. **(D)** Whole-cell lysates were analyzed by Western blot analysis of the indicated proteins. The experiments were performed in triplicate (*p < 0.05; **p < 0.01; and ***p < 0.001).

To investigate whether LPC enhances phagosome maturation during Mtb infection in mouse macrophages, maturation of phagosomes containing FITC-labeled Mtb was monitored by measuring the colocalization of several markers. As shown in Figure 3A, the early phagosome marker, EEA1, and the late phagosome marker, lysosomal-associated membrane protein 1 (LAMP-1), showed a consistently increased ratio of colocalization with FITC-labeled Mtb-containing phagosomes throughout H37Ra or H37Rv infection. In addition, the colocalized expression ratio of another early phagosomal marker, Ras-associated protein 5 (Rab5), and late phagosomal marker, Rab7, with Mtb immediately increased within 30 min when cells were stimulated with LPC (Figure S2A in Supplementary Material). Furthermore, over the course of Mtb infection, Rab5 and Rab7 showed similar kinetics to those observed for EEA1 and LAMP-1.

Phosphatidylinositol 3 kinase regulates Rab5 and Rab7 recruitment to the phagosome as well as phagosome formation and maturation (11). The above results have shown that LPC treatment activates PI3K–p38 MAPK and controls Mtb growth in H37Ra-infected cells (Figures 1 and 2). To determine whether inhibition of the PI3K signaling pathway affects mycobacterial survival within macrophages and phagosome maturation in the presence of LPC, Raw264.7 cells were pretreated with the PI3K inhibitor, wortmannin, and bacterial CFU were measured. First, we confirmed that Raw264.7 cells exposed to wortmannin alone did not affect cell viability (data not shown). Intracellular Mtb survival was decreased in LPC-treated cells, whereas wortmannin did not restrict the replication of H37Ra in LPC-treated cells (Figure 3B). In H37Ra-infected and LPC-treated macrophages, the highly increased ratio of Rab5 colocalized with Mtb clearly decreased to a level similar to that in Mtb-infected macrophages when PI3K signaling was abolished with wortmannin (Figure 3C).

Several proteases in the cathepsin family have bactericidal effects, such as cathepsin B, D, G, and L (31). The phagosomal proteases cathepsin D and cathepsin L kill *Mycobacterium avium* and *L. monocytogenes*, indicating that several cathepsins have antimicrobial activity (32, 33). In particular, cathepsin D is a major intracellular aspartic protease of endosomes and lysosomes that is synthesized on the rough endoplasmic reticulum as a prepro-enzyme that undergoes several proteolytic cleavages to produce the mature form depending on the action of cysteine lysosomal and/or aspartic proteases at acidic pH (34). Therefore, to further investigate whether phagosome maturation is promoted by the treatment of LPC in Mtb-infected cells, the expression level of cleaved cathepsin D was detected in H37Ra-infected cells. As shown in Figure 3D, the expression level of cleaved cathepsin D rapidly increased in LPC-treated cells over the course of Mtb infection compared with its level in untreated or uninfected cells. Furthermore, although cells were stimulated with LPC, the inhibition of PI3K with wortmannin delayed the cleavage of cathepsin D in Mtb-infected cells (Figure 3D). By contrast, pretreatment with bafilomycin A, a specific inhibitor of the vacuolar H^+^-ATPase, perturbed phagosome acidification and decreased the conversion into the mature form of cathepsin D in H37Ra-infected cells in the presence of LPC (Figure S3A in Supplementary Material). We also found that bafilomycin A1 had no effect on cell viability as determined by a trypan blue exclusion assay (data no shown). Moreover, FITC-labeled H37Ra-containing phagosomes showed a substantially increased level of colocalization with Rab5 or Rab7 in LPC-treated cells during Mtb infection, whereas bafilomycin A decreased the amount of Rab5 or Rab7 associated with FITC-labeled H37Ra-containing phagosomes (Figure S3B in Supplementary Material). To verify phagosome acidification during the process of maturation, cells were stained with LysoSensor DND-160. The green fluorescence intensity was significantly increased in H37Ra-infected macrophages followed by LPC treatment (Figure S3C in Supplementary Material). These data indicate that LPC promotes phagosomal maturation, which ultimately leads to phagolysosome fusion and bacterial degradation in macrophages during H37Ra infection.

### LPC Enhances Phagosomal Maturation Through the PI3K–p38 MPAK Signaling Pathway

**Figure 4:**
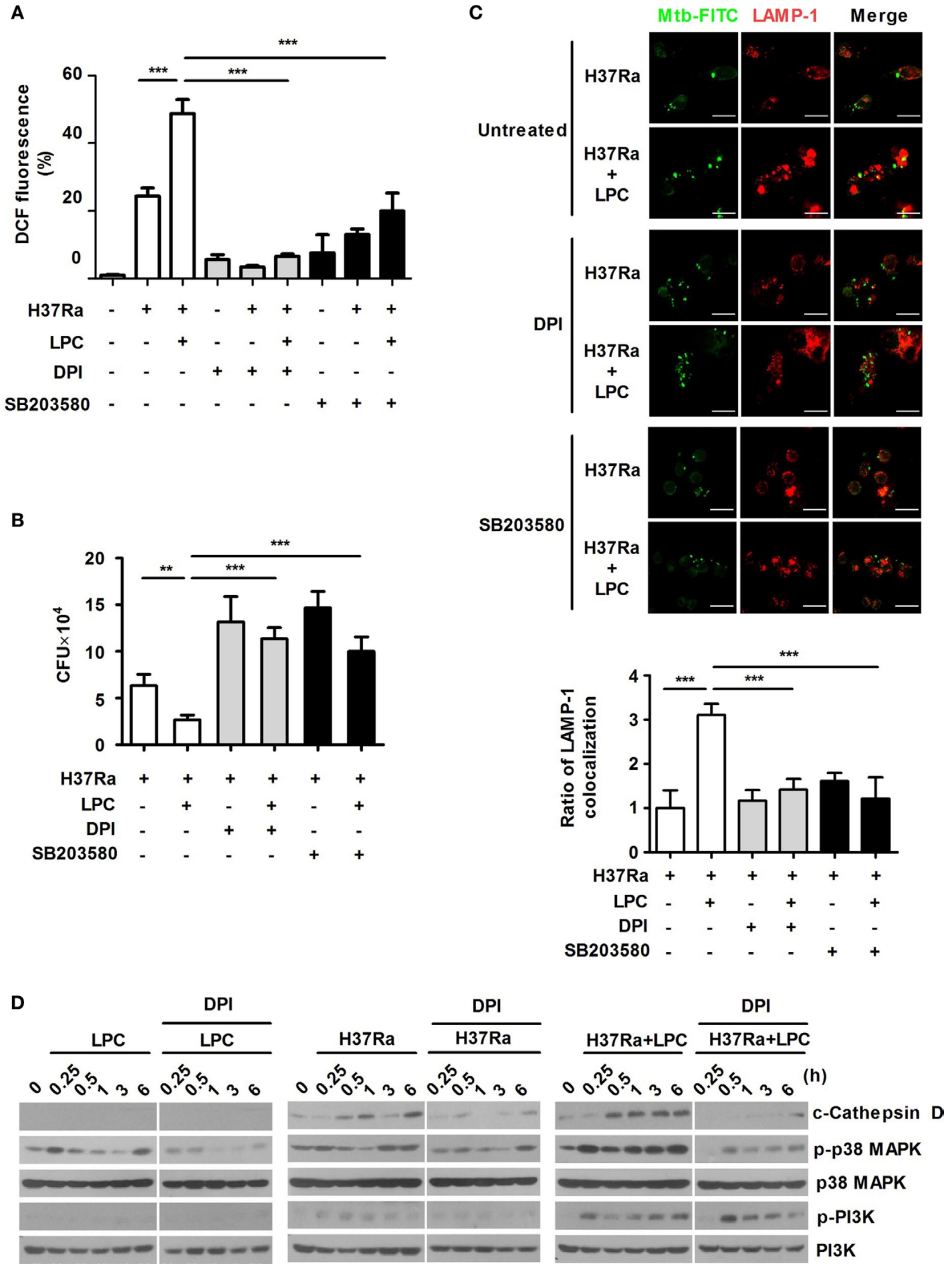
p38 mitogen-activated protein kinase (MAPK) and reactive oxygen species contribute to phagosome maturation in lysophosphatidylcholine (LPC)-treated macrophages during *Mycobacterium tuberculosis* (Mtb) infection. **(A)** Raw264.7 cells were pretreated with SB203580 (10 µM) or diphenyleneiodonium (DPI) (10 µM) for 1 h and then stimulated with LPC during H37Ra infection [multiplicity of infection (MOI) of 5]. After infection, cells were labeled with 2′,7′-dichlorofluorescein diacetate and then measured by confocal microscopy. The bar graph represents the percentage of dihydrodichlorofluorescein (DCF) fluorescence. **(B)** The growth of intracellular bacteria was determined by colony-forming unit (CFU) in LPC-treated Raw264.7 cells in the presence or absence of each inhibitor. **(C)** Raw264.7 cells were pretreated with SB203580 (10 µM) or DPI (10 µM) for 1 h and then stimulated with LPC during H37Ra (MOI of 5) infection. After infection, cells were fixed and stained with lysosomal-associated membrane protein 1 (LAMP-1). All images were obtained by confocal microscopy. The bar graphs represent the ratio of Mtb colocalization with LAMP-1. **(D)** Whole-cell lysates were analyzed by Western blot analysis of the indicated proteins (**p < 0.01 and ***p < 0.001).

To assess whether LPC-induced activation of p38 regulates ROS production and bacterial growth, Raw264.7 cells were pretreated with the ROS scavenger DPI or the p38 inhibitor SB203580. We initially confirmed that these inhibitors did not decrease cell survival (data not shown). As shown in Figure 4A and Figures S4A,B in Supplementary Material, inhibition of p38 MAPK suppressed ROS and NO production in LPC-treated cells during Mtb infection. LPC treatment also significantly decreased intracellular Mtb growth, but inhibition of ROS production or the p38 MAPK pathway was sufficient to recover bacterial growth in LPC-treated cells during H37Ra infection (Figure 4B). These data suggest that LPC strongly induces production of ROS and controls intracellular Mtb growth by enhancing the p38 MAPK signaling pathway.

TLR signaling mediates the control of phagosome maturation through the adaptor MyD88 and p38 MAPK (30). To investigate whether p38 MAPK contributes to the enhanced phagosome maturation caused by LPC in Mtb-infected macrophages, Raw264.7 cells were pretreated with SB203580. The early phagosome marker EEA1 showed strong colocalization with Mtb during the early phase of infection, whereas inhibition of p38 MAPK with SB203580 reduced the colocalization of EEA1 with Mtb in LPC-treated cells (Figure S4C in Supplementary Material). Furthermore, LPC treatment increased LAMP-1 colocalization with Mtb; however, inhibition of p38 MAPK caused a twofold decrease in Mtb-containing phagosome colocalization with LAMP-1 in LPC-treated cells (Figure 4C). Moreover, the LPC-induced increase in EEA1 or LAMP-1 colocalization with FITC-labeled H37Ra was reduced by DPI treatment (Figure S4C in Supplementary Material; Figure 4C). DPI inhibited LPC-induced processing of cathepsin D and phosphorylated p38 MAPK; however, the level of phosphorylated PI3K was not influenced by DPI treatment (Figure 4D). These findings indicate that phagosome maturation is regulated by LPC-induced p38 MAPK and ROS during the early phase of infection.

### LPC Increases Cytosolic Ca^2+^ Release *via* the PI3K–p38 MAPK Signaling Pathway in Mtb-Infected Macrophages

**Figure 5:**
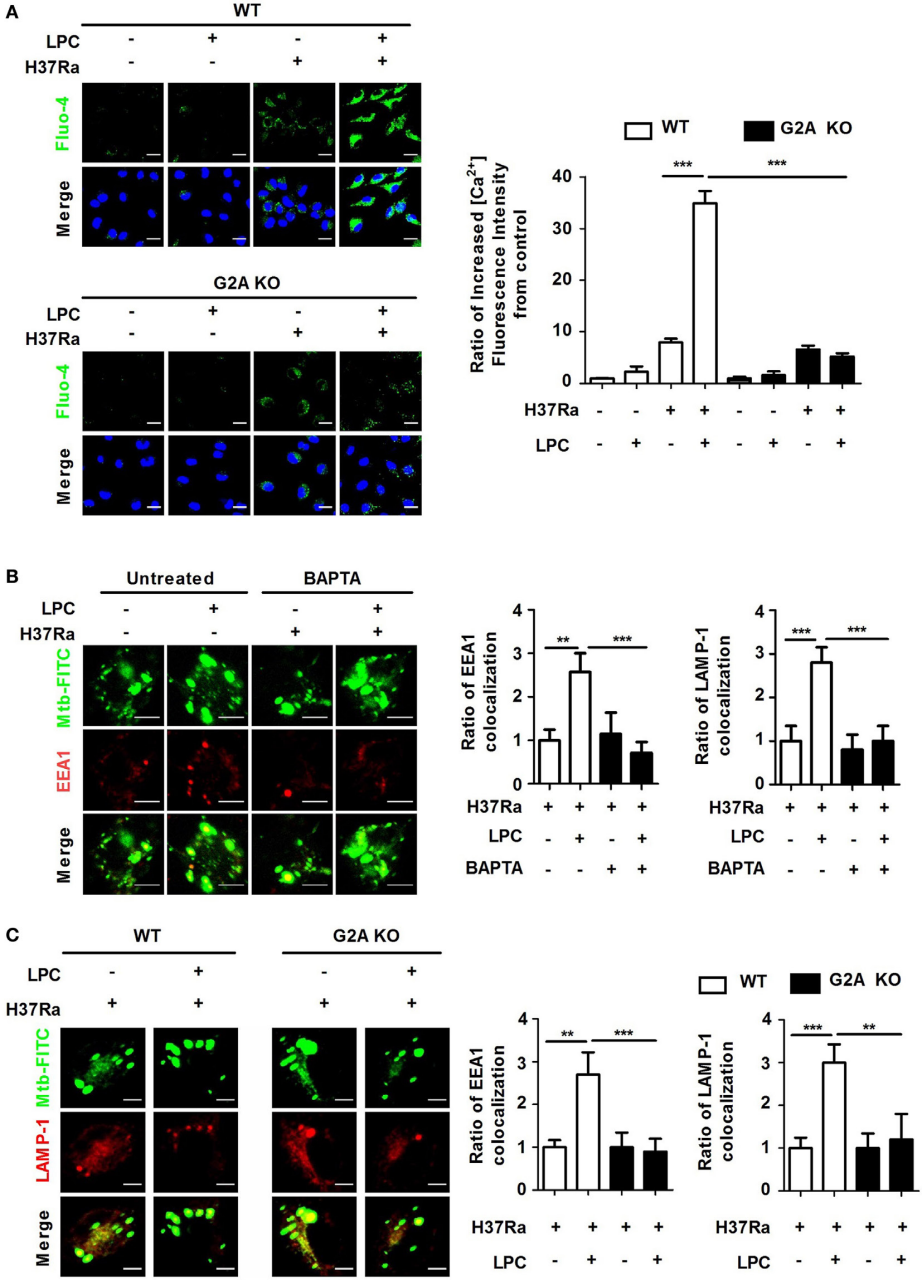
Loss of G2A does not induce enhanced phagosome maturation or intracellular Ca^2+^ release in lysophosphatidylcholine (LPC)-treated macrophages during H37Ra infection. **(A)** Wild-type (WT) and G2A knockout (KO) bone marrow-derived macrophages (BMDMs) were infected with H37Ra [multiplicity of infection (MOI) of 5] and treated with LPC for 30 min. Cells were then loaded with calcium sensing Fluo-4/AM and stained with diamidino-2-phenylindole to identify nuclei. All images were observed by confocal microscopy. The bar graph represents the ratio of the Fluo-4/AM mean fluorescence intensity (MFI), which was normalized to the MFI obtained for uninfected cells. **(B)** Raw264.7 cells were pretreated with BAPTA/AM (30 µM) for 30 min and stimulated with LPC during fluorescein isothiocyanate (FITC)-labeled H37Ra infection (MOI of 5). After infection, cells were fixed and stained with early endosomal antigen 1 (EEA1) and lysosomal-associated membrane protein 1 (LAMP-1). The bar graphs represent the ratio of *Mycobacterium tuberculosis* (Mtb) colocalization with each marker. **(C)** WT and G2A KO BMDMs were infected with FITC-labeled H37Ra with or without LPC treatment for 3 h. After infection, cells were fixed and stained with EEA1 and LAMP-1. All images were viewed under a confocal microscope. The bar graphs represent the ratio of Mtb colocalization with each marker (**p < 0.01 and ***p < 0.001).

Stossel has shown that cytosolic Ca^2+^ regulates subsequent steps in the phagocytic process and phagosomal maturation (35). To investigate whether Ca^2+^ is required for LPC-induced phagosome maturation during Mtb infection, cells were loaded with the Ca^2+^-sensing probe, Fluo-4/AM. Compared with untreated Mtb-infected cells, Mtb-infected cells treated with LPC showed a rapid increase in intracellular Ca^2+^ fluorescence after 30 min of infection (Figure 5A). However, the accelerated accumulation of intracellular Ca^2+^ was not detected in G2A KO macrophages when cells were infected with Mtb followed by LPC treatment. To further explore whether cytosolic Ca^2+^ release *via* the PI3K–p38 MAPK signaling pathway contributes to LPC-mediated phagosome maturation during Mtb infection, cells were pretreated with the calcium chelator BAPTA/AM, which can be used to control the level of intracellular Ca^2+^. Pretreatment with 30 µM BAPTA/AM was not cytotoxic to Raw264.7 cells (data not shown). As shown in Figure 5B and Figure S5B in Supplementary Material, the LPC-induced increase in EEA1 or LAMP-1 colocalization with FITC-labeled H37Ra-containing phagosomes was significantly reduced in Mtb-infected cells after treatment with BAPTA/AM to a similar level to that in G2A KO cells (Figure S5A in Supplementary Material). G2A KO macrophages also exhibited reduced accumulation of EEA1 and LAMP-1 expression in Mtb-containing vacuoles (Figure 5C). In addition, BAPTA/AM only partially reduced processing of cathepsin D in LPC-treated cells during Mtb infection; however, the level of phosphorylated PI3K and p38 remained unchanged by BAPTA/AM (Figure S5D in Supplementary Material). Finally, when cells were pretreated with the ROS scavenger apocynin or the PI3K inhibitor wortmannin, intracellular calcium decreased (Figure S5D in Supplementary Material). These observations suggest that intracellular ROS production and the PI3K pathway are required for intracellular Ca^2+^ release in LPC-treated cells during the early phase of Mtb infection.

### Elevation of Intracellular cAMP Influences LPC-Induced ROS Production and Phagosome Maturation During Mtb Infection

**Figure 6:**
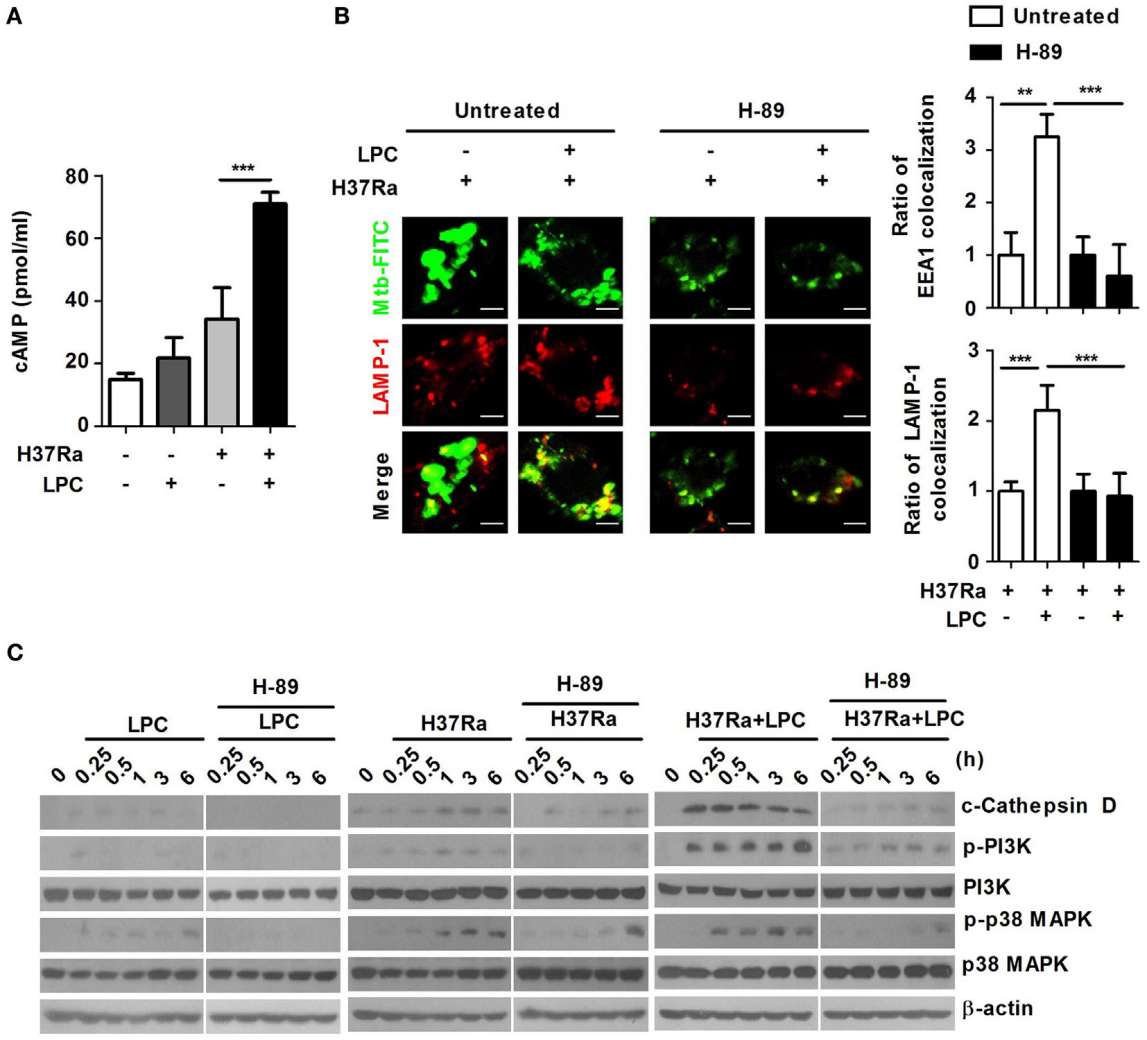
Intracellular cyclic adenosine monophosphate (cAMP) influences phagosome maturation *via* protein kinase A (PKA)–phosphatidylinositol 3 kinase (PI3K)–p38 mitogen-activated protein kinase (MAPK) signaling in lysophosphatidylcholine (LPC)-treated macrophages during *Mycobacterium tuberculosis* (Mtb) infection. **(A)** Raw264.7 cells were infected with H37Ra [multiplicity of infection (MOI) of 5] and treated with LPC for 15 min. Cell lysates were collected, and cAMP levels were measured using a specific enzyme immunoassay kit. **(B)** Raw264.7 cells were pretreated with H-89 (10 µM) for 1 h and infected with fluorescein isothiocyanate (FITC)-labeled H37Ra (MOI of 5) with or without LPC treatment for 3 h. After infection, the cells were fixed and stained with early endosomal antigen 1 (EEA1) and lysosomal-associated membrane protein 1 (LAMP-1). Mtb colocalization with each phagosome maturation marker was observed by confocal microscopy. The bar graphs represent the ratio of Mtb colocalization with each marker. **(C)** Whole-cell lysates were analyzed by Western blot analysis of the indicated proteins.

Cyclic adenosine monophosphate can also function as a positive regulator of several immune functions to activate immune cells (36, 37). Suppression of LPS-induced cytokine production by cAMP is mediated by PKA-dependent activation of the NF-κB pathway in macrophages (38). To determine the effects of cAMP on LPC-induced phagosome maturation in Mtb-infected macrophages, the intracellular cAMP level was observed in LPC-treated cells. During Mtb infection, the intracellular cAMP level was significantly higher in LPC-treated than in untreated Mtb-infected cells (Figure 6A).

The primary mediator of the intracellular response to cAMP is cAMP-dependent kinase (PKA) (39). Therefore, to identify whether the elevated level of intracellular cAMP could trigger production of ROS and NO by activating PKA in LPC-treated cells during Mtb infection, cells were pretreated with H-89, a PKA inhibitor that had no effect on cell viability (data not shown). Inhibition of PKA did not induce production of ROS or NO in LPC-treated cells during Mtb infection compared with that in untreated Mtb-infected cells (Figures S6B,C in Supplementary Material). These results suggest that LPC-induced enhancement of intracellular cAMP triggers production of ROS and NO through a PKA-dependent signaling pathway in macrophages during Mtb infection. LPC treatment induced a large fluorescence signal from the Ca^2+^-sensing probe, Fluo-4/AM; however, inhibition of PKA with H-89 markedly decreased LPC-induced enhancement of the cytosolic Ca^2+^ release in Mtb-infected cells, indicating that LPC enhanced cytosolic Ca^2+^ release through the cAMP-dependent PKA signaling pathway during Mtb infection (Figure S6D in Supplementary Material).

To assess the effect of intracellular cAMP levels on Mtb-containing phagosome maturation in LPC-treated cells, Mtb-containing phagosomes were examined for EEA1 and LAMP-1 recruitment in the presence or absence of H-89. As shown in Figure 6B and Figure S6A in Supplementary Material, inhibition of PKA with H-89 caused a significant decrease in Mtb-containing phagosome colocalization with EEA1 and LAMP-1 in LPC-treated cells. Furthermore, the expression level of cleaved cathepsin D rapidly increased in LPC-treated cells over the course of Mtb infection, whereas inhibition of PKA reduced the conversion into the mature form of cathepsin D in H37Ra-infected cells in the presence of LPC (Figure 6C). Notably, inhibition of PKA with H-89 resulted in decreased phosphorylation of PI3K and p38 MAPK in LPC-treated cells during Mtb infection. Taken together, these findings suggest that LPC induces enhanced progression of phagosome maturation through cAMP-dependent activation of the PKA–PI3K–p38 MAPK signaling pathway during Mtb infection.

### LPC Regulates Phagosome Maturation *via* a PLC-Induced Increase in Cytosolic Ca^2+^ Release in Mtb-Infected Macrophages

**Figure 7:**
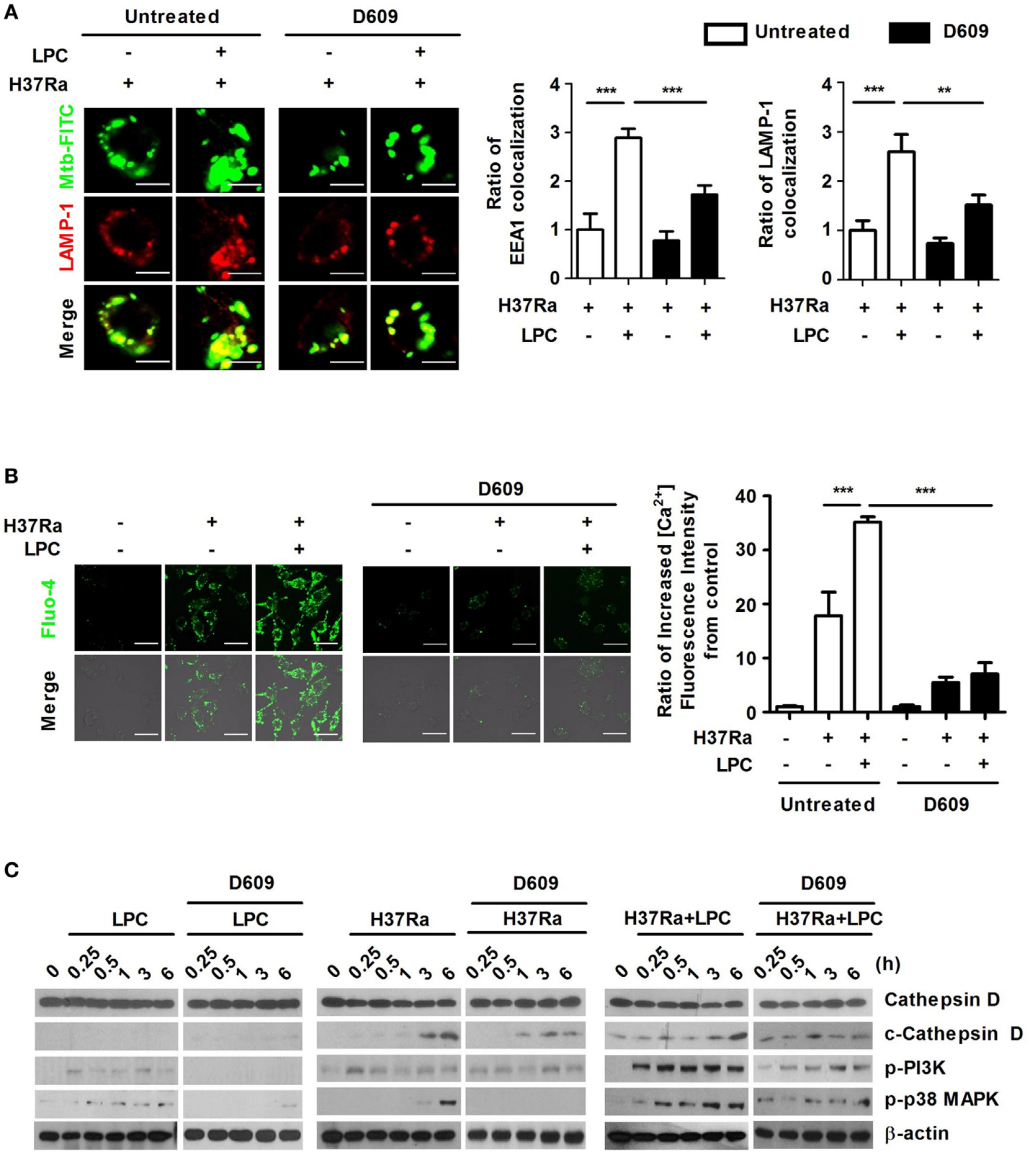
Lysophosphatidylcholine (LPC) enhances phagosome maturation by increasing cytosolic Ca^2+^ release through G2A-mediated activation of phospholipase C during H37Ra infection. **(A)** Raw264.7 cells were pretreated with D609 (10 µM) for 12 h and infected with H37Ra (multiplicity of infection of 5) with or without LPC treatment for 3 h. *Mycobacterium tuberculosis* (Mtb) colocalization with early endosomal antigen 1 (EEA1) or lysosomal-associated membrane protein 1 (LAMP-1) was observed by confocal microscopy. The bar graphs represent the ratio of Mtb colocalization with each marker. **(B)** Cells were loaded with Fluo-4/AM and stained with the nuclear dye diamidino-2-phenylindole. Intracellular Ca^2+^ release was observed by confocal microscopy. The bar graph represents the ratio of Fluo-4/AM mean fluorescence intensity (MFI), which was normalized to the MFI obtained for uninfected cells. **(C)** Whole-cell lysates were analyzed by Western blot analysis of the indicated proteins (**p < 0.01 and ***p < 0.001).

The G2A receptor strongly activates PLC and induces elevated intracellular Ca^2+^ levels, ERK, Rho, and Rac activation (40). Therefore, to identify whether G2A-mediated activation of PLC could regulate the process of phagosome maturation and the levels of intracellular Ca^2+^ in LPC-treated cells during Mtb infection, cells were pretreated with D609, a PLC inhibitor (a competitive inhibitor of PC-PLC) and then stimulated the cells with LPC together with Mtb infection. D609 treatment did not affect cell viability (data not shown) and markedly decreased the colocalization of EEA1 or LAMP-1 with Mtb-containing vacuoles (Figure 7A; Figure S7A in Supplementary Material). LPC treatment induced and increased the level of intracellular Ca^2+^ fluorescence in LPC-treated cells, but PLC inhibition markedly decreased the fluorescence signal of intracellular Ca^2+^ during Mtb infection (Figure 7B). Furthermore, inhibition of PLC only partially reduced the expression level of cleaved cathepsin D in LPC-treated cells during Mtb infection; however, the level of phosphorylated PI3K and p38 was significantly reduced by D609 treatment (Figure 7C). These results suggest that LPC-induced activation of PLC-mediated signaling (Figure S8 in Supplementary Material) mediates phagosomal maturation during Mtb infection in macrophages.

### Cytosolic Ca^2+^ Modulates the LPC-Induced Inflammatory Response by Regulating Phosphorylation of GSK3β in Mtb-Infected Macrophages

**Figure 8:**
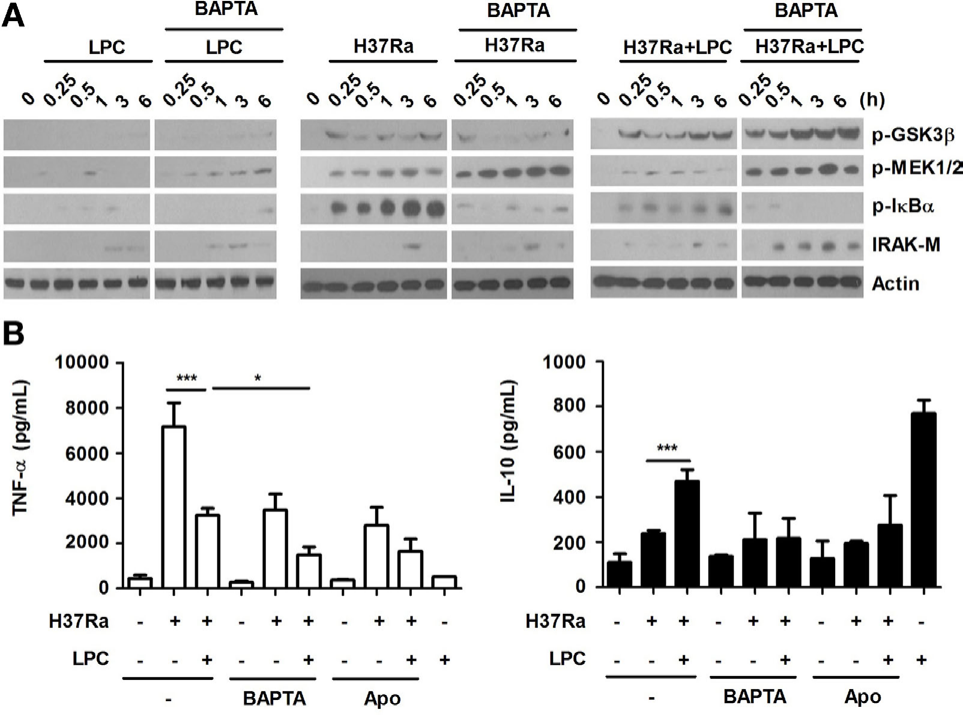
Lysophosphatidylcholine (LPC)-induced intracellular Ca^2+^ release and reactive oxygen species production regulate activation of NF-κB signaling and production of inflammatory cytokines by modulating phosphorylation of glycogen synthase kinase 3 beta (GSK3β) (Ser 9) in H37Ra-infected macrophages. **(A)** Raw264.7 cells were pretreated with BATPA/AM (30 µM) and stimulated with LPC during H37Ra infection (multiplicity of infection of 5). Whole-cell lysates were analyzed by Western blot analysis of the indicated proteins. **(B)** The production of TNF-α and IL-10 was measured 24 h post-infection in the presence or absence of BAPTA/AM (BAPTA) or apocynin (Apo) (*p < 0.05 and ***p < 0.001).

Glycogen synthase kinase 3 beta differentially regulates pro- and anti-inflammatory cytokine responses in *F. tularensis* LVS-stimulated macrophages (41). To determine whether LPC regulates the inflammatory response through cytosolic Ca^2+^, PI3K and its downstream signaling components, such as Akt and GSK3β, phosphorylation of GSK3β, and activation of signaling components involving the NF-κB pathway were detected in LPC-treated cells during Mtb infection. Chelation of cytosolic Ca^2+^ induced increased levels of IRAK-M, leading to a decreased level of phosphorylated IκBα in LPC-treated cells during H37Ra infection (Figure 8A). LPC treatment also significantly enhanced phosphorylation of PI3K and GSK3β in H37Ra-infected cells (Figure 8A). BAPTA/AM treatment completely blocked production of TNF-α by LPC-treated cells without decreasing IL-10 production (Figure 8B). Furthermore, pretreatment with apocynin significantly inhibited production of TNF-α, but not IL-10, in LPC-treated cells during Mtb infection. These findings suggest that cytosolic Ca^2+^ mediates phosphorylation of GSK3β to modulate the NF-κB pathway. Taken together, these observations indicate that GSK3β signaling inhibits excessive inflammation through both decreased pro-inflammatory cytokine production and increased anti-inflammatory cytokine IL-10 production.

### LPC Regulates the Expression of Inflammatory Cytokines by Enhancing Phosphorylation of GSK3β *via* the Intracellular cAMP-Dependent PKA Signaling Pathway

**Figure 9:**
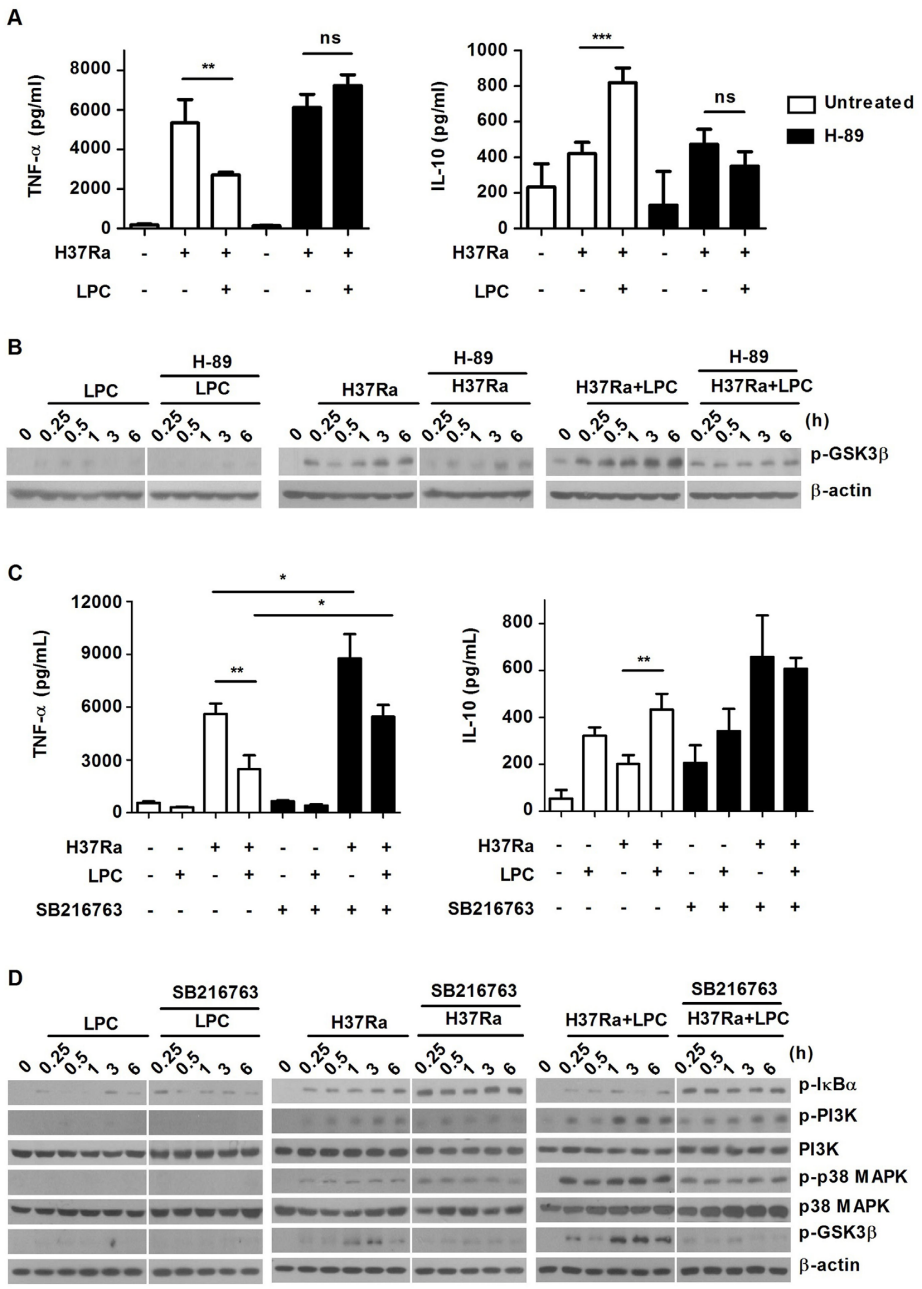
Protein kinase-mediated signaling is involved in production of inflammatory cytokines by modulating phosphorylation of glycogen synthase kinase 3 beta (GSK3β) (Ser 9) in H37Ra-infected macrophages. **(A)** Raw264.7 cells were pretreated with H-89 for 1 h and then stimulated with lysophosphatidylcholine (LPC) during H37Ra infection [multiplicity of infection (MOI) of 5]. Production of TNF-α and IL-10 was measured in the cell culture medium at 24 h. **(B)** Whole-cell lysates were analyzed by Western blot analysis of the indicated proteins. **(C)** Raw264.7 cells were pretreated with SB216763 (10 µM) for 1 h and then stimulated with LPC during H37Ra infection (MOI of 5). Production of TNF-α and IL-10 was measured by enzyme-linked immunosorbent assay at 24 h. **(D)** Whole-cell lysates were analyzed by Western blot analysis of the indicated proteins; *p < 0.05; **p < 0.01; ***p < 0.001, and not significant (ns) (*p* > 0.05).

To further examine whether the intracellular cAMP-dependent PKA signaling pathway is associated with production of inflammatory cytokines by modulating GSK3β, cytokine production was measured in the presence or absence of H-89. Compared with untreated Mtb-infected macrophages, treated with LPC exhibited a significant decrease in TNF-α production and an increase in IL-10 expression (Figure 9A). However, pretreatment with H-89 strikingly enhanced TNF-α production and suppressed IL-10 production. To elucidate the association between PKA and GSK3β, phosphorylation of GSK3β was examined in H-89-pretreated cells. As shown in Figure 9B, LPC induced increased phosphorylation of GSK3β, but H-89 treatment significantly impeded phosphorylation of GSK3β induced by LPC during Mtb infection. Taken together, these data suggest that increased phosphorylation of GSK3β modulates pro-inflammatory cytokine production by suppressing the NF-κB signaling pathway in the presence of LPC during Mtb infection.

To further assess whether PLC modulates inflammatory responses by activating the G2A-mediated signaling pathway during Mtb infection, production of inflammatory cytokines was measured in supernatants during Mtb infection. Phosphorylated GSK3β was markedly increased according to inhibition of PLC in LPC-treated cells during H37Ra infection (Figure S7B in Supplementary Material). Consistent with this finding, inhibition of PLC also induced decreased production of inflammatory mediators, including TNF-α and IL-10, in LPC-treated cells during Mtb infection (Figure S7C in Supplementary Material). These data suggest that LPC regulates inflammatory responses by increasing phosphorylation of GSK3β through G2A-mediated activation of PLC during Mtb infection.

Next, to further assess the effects of GSK3β on inflammatory responses during Mtb infection, production of pro- and anti-inflammatory cytokines was detected in the presence and absence of the GSK3β inhibitor SB216763. SB216763 is a highly specific GSK3 inhibitor that dose not affect cell proliferation (data not shown). As shown in Figure 9C, LPC treatment increased the level of TNF-α and decreased the level of IL-10. In addition, GSK3β inhibition with SB216763 partially enhanced production of TNF-α, while production of IL-10 remained unchanged in LPC-treated cells during Mtb infection. Similarly, NO production was significantly increased in LPC-treated cells during Mtb infection; however, it was slightly reduced by GSK3β inhibition (data not shown). In addition, LPC markedly decreased the level of phosphorylated IκBα according to the increased phosphorylation of GSK3β, whereas inhibition of GSK3β with SB216763 restored the phosphorylated level of IκBα, in accordance with the decreased phosphorylation of GSK3β (Figure 9D). These results demonstrate that GSK3β negatively modulates the LPC-induced inflammatory response *via* inhibition of the NF-κB signaling pathway in Mtb-infected macrophages (Figure S8 in Supplementary Material).

## Discussion

*Mycobacterium tuberculosis* can regulate and interfere with innate and adaptive immune responses to survive and persist in host cells. Mtb also has several strategies to evade the host immune system, such as inhibition of phagosome maturation, phagosome acidification, and generation of microbicidal agents, including pro-inflammatory cytokines, chemokines, ROS, and reactive nitrogen intermediates (42). Although many studies have attempted to define the interaction between Mtb and the macrophage phagosome, very little is known about the signaling events in host cells. Therefore, it is important to understand the signaling pathways in macrophages in response to Mtb infection and whether Mtb modulates these pathways.

Lysophosphatidylcholine, an endogenous phospholipid, has various stimulating or modulating effects on immune cells, such as monocytes/macrophages, neutrophils, and lymphocytes (28). Most studies have demonstrated pro-inflammatory activities of LPC; however, LPC possesses anti-inflammatory properties. Systemic administration of LPC protects mice against experimental sepsis-induced lethality, enhances bacterial clearance, and inhibits the production of pro-inflammatory cytokines, including TNF-α and IL-1β (20). In addition, LPC enhanced the uptake process of *L. major* in DCs and regulated production of TNF-α and NO (23). Since excessive inflammation triggers host damage during infection with pathogens, LPC is an attractive target for development of a therapeutic agent. In TB patients, the concentration of LPC in the serum was significantly lower than that in healthy donors (43, 44). In addition, Mtb induces macrophage apoptosis through inhibiting phospholipase A_2_, which may offer a reasonable explanation for the lower level of LPC in the serum of TB patients (43). Therefore, the concentration of LPC in the serum could be developed as a useful diagnostic marker in TB. Moreover, LPC could directly kill drug-resistant bacteria *via* inducing bacterial membrane depolarization (21) and could be an effective adjuvant in treatment of drug-resistant pneumonia patients infected with *Acinetobacter baumannii* (45). The recent finding that malaria parasites can sense and process host-derived LPC (24) to regulate their differentiation stages provides a very important insight into the interaction between host and pathogen.

In this study, compared with untreated Mtb-infected macrophages, Mtb-infected macrophages treated with LPC exhibited reduced intracellular bacterial growth during Mtb infection. In addition, LPC treatment allowed phagosome maturation innate responses to proceed more effectively in Mtb-infected macrophages through a marked increase in the levels of the early endosomal markers Rab5 and EEA1 and late phagosomal markers Rab7 and LAMP-1 associated with Mtb-containing phagosomes. The mouse macrophage Raw264.7 cell line and primary BMDMs treated with LPC displayed promotion of phagosome maturation according to the increased expression level of mature cathepsin D after Mtb H37Ra infection. LPC also significantly increased secretion of antimicrobial agents, such as ROS and NO, and the anti-inflammatory cytokine IL-10, whereas it markedly decreased secretion of the pro-inflammatory cytokine TNF-α in Mtb-infected macrophages. These data suggest that LPC directly regulates early macrophage responses without excessive production of inflammatory mediators after Mtb infection.

Cyclic adenosine monophosphate is an important second messenger and regulates various physiological processes. However, because host cAMP levels represent a major virulence strategy adopted by several pathogens, a direct role of cAMP in mycobacterial pathogenesis has not been established (42). The cAMP signaling inhibitor H-89 (PKA inhibitor) increases the survival of Mtb H37Rv in J774 macrophages, suggesting that intracellular cAMP levels determine mycobacterial survival by regulating phagolysosome fusion and phagosome acidification in host macrophages (46). However, the role of cAMP in host macrophages infected with *Mycobacteria* remains poorly understood. This study showed that the levels of intracellular cAMP markedly increased in LPC-treated cells during Mtb infection; by contrast, inhibition of PKA markedly decreased Mtb-phagosome maturation and production of ROS and cytosolic Ca^2+^ in LPC-treated cells. Our data suggest that LPC regulates Mtb-phagosome maturation *via* elevated intracellular cAMP levels.

Phosphatidylinositol 3 kinase activity regulates phagosome maturation downstream of numerous phagocytic receptors, and it involves activation and recruitment of various small Rab GTPases that modulate endosomal trafficking. The TB toxin lipoarabinomannan (LAM) arrests phagosome maturation through the Ca^2+^/calmodulin-PI3K hVPS34 cascade in macrophages (47). By contrast, another group demonstrated that phosphatidylinositol mannoside, a biosynthetic precursor of LAM, stimulates Rab5 to enhance early endosomal fusion in a PI3K-independent manner in macrophages (48). These reports suggest that PI3K is involved in modulation of phagosome maturation according to different stimuli. p38 MAPK signaling plays an important role in membrane trafficking and phagosome maturation. In addition, inhibition of p38 MAPK promotes *Mycobacteria*-containing phagosome maturation through decreased IL-10 production accompanied by increased IL-12 (49). Activated p38 MAPK enhances GDP dissociation inhibitor (GDI) removal of Rab5 from the membrane upon GDI phosphorylation, leading to the displacement of EEA1 from endosomal membranes (50). This report suggests that the effect of phospho-p38 MAPK on the association of EEA1 with membranes is likely due to blockade of the binding of EEA1 to Rab5. This study showed that LPC treatment significantly increased the expression level of phosphorylated PI3K and p38 MAPK in H37Ra-infected macrophages. However, this study confirmed the decrease in Mtb-phagosome maturation, ROS, and Ca^2+^ production in LPC-treated cells during inhibition of the PI3K–p38 MAPK signaling pathway. These results suggest that LPC exerts its effects on Mtb-phagosome maturation in a manner that is dependent on the PKA–PI3K–p38 MAPK signaling pathway.

Increased cytosolic Ca^2+^ release regulates subsequent steps in the phagocytic process and is required for efficient phagosome maturation (7). Several other studies have suggested that increased cytosolic Ca^2+^ mediates phagosome maturation (35, 51). In addition, Mtb modulates Ca^2+^-dependent processes to evade phagocytic killing (13). These results revealed the potential role of cytosolic Ca^2+^ release in Mtb-phagosome maturation in macrophages. Treatment with LPC resulted in increased cytosolic Ca^2+^ release that enhanced Mtb-containing phagosome maturation. However, the intracellular Ca^2+^ chelator, BAPTA/AM, abolished the LPC-induced increase in phagosome maturation in Mtb-infected macrophages. These results suggest that LPC mediates the regulation of Mtb-phagosome maturation through cytosolic Ca^2+^ release in macrophages.

Phospholipase C generates inositol triphosphate (IP_3_) and diacylglycerol (DAG) by cleaving PI(4,5)P_2_ and then induces release of the second messenger Ca^2+^ from intracellular stores (7). Since PLC-dependent Ca^2+^ signals are triggered during particle binding, Ca^2+^ signals might occur during the early stage of the phagocytic process (12). This study also showed that pretreatment with D609, a PLC inhibitor, blocked IP_3_ and DAG generation and inhibited phagosome maturation by decreasing intracellular Ca^2+^ release in LPC-treated cells during the early stages of H37Ra infection. In addition, inhibition of PLC with D609 reduced production of inflammatory mediators, including TNF-α, IL-10, and NO, in LPC-treated cells during H37Ra infection. These results also suggest that LPC mediates regulation of Mtb-phagosome maturation and inflammatory mediators through the PLC-induced release of cytosolic Ca^2+^ in macrophages.

GSK3 is a multifunctional Ser/Thr kinase with two isoforms, GSK3α and GSK3β, and it is regulated by phosphorylation for activation and inhibition, leading to regulation of the innate and adaptive immune systems. Inactivation of GSK3β by Akt-induced phosphorylation may result in activation of transcription factors during bacterial infection, including AP-1, STAT-1, STAT-3, and NF-κB. However, GSK3 plays distinct roles in regulation of NF-κB depending on the physiological state of the cell (52). PI3K–Akt-dependent inhibition of GSK3β differentially affects the nature and magnitude of the inflammatory response in LPS-stimulated human monocytes (53). Furthermore, GSK3β activity negatively regulates the levels of the anti-inflammatory cytokine IL-1Ra in LPS-stimulated human monocytes (54). A study utilizing *Mycobacterium bovis* BCG also demonstrated that inhibition of GSK3β through PI3K–Akt signaling increases production of IL-10 in primary human blood monocytes (55). In agreement with this finding, this study showed that LPC treatment significantly enhanced the level of phosphorylated GSK3β (Ser9), whereas it induced decreased phosphorylation of IκBα during Mtb infection. This study also demonstrated that inhibition of GSK3β with a pharmacologic inhibitor restored production of TNF-α together with the level of phosphorylated IκBα in LPC-treated cells. These findings support the hypothesis that LPC might modulate the inflammatory response by increasing phosphorylation of GSK3β *via* a PKA-mediated pathway during Mtb infection.

During Mtb infection, a sufficient level of inflammatory response contributes to limit bacterial growth, but it also induces tissue damage in the host. Macrophages infected with Mtb produce inhibitory cytokines such as TGF-β and IL-10, which reduces macrophage activation, thereby leading to decreased bacterial clearance (56, 57). However, IL-10 deficiency in mice has no protective effect during Mtb infection (58, 59). Thus, the role of IL-10 in Mtb infection remains controversial. To assess whether low production of pro-inflammatory cytokines was due to elevated levels of IL-10 production in LPC-treated cells during Mtb infection, we isolated BMDMs from IL-10 KO mice and infected them with Mtb. During Mtb infection, IL-10 KO BMDMs also controlled Mtb growth to a level similar to that in WT BMDMs treated with LPC (data not shown). In addition, production of pro-inflammatory cytokines decreased in IL-10 KO BMDMs after treatment with LPC. Therefore, LPC could help macrophages to control infection without excessive inflammatory cytokine production either in the absence or presence of IL-10 production.

In this study, we demonstrated how LPC could simultaneously control Mtb growth and regulate excessive production of pro-inflammatory cytokines in macrophages on a molecular basis. Therefore, LPC may constitute a potential target for development of therapeutic agents for treatment of TB.

## Ethics Statement

This study was managed in strict accordance with the Guide for the Care and Use of Laboratory Animals (National Research Council, 2011) and all experimental animals procedures used in this study were handled using a protocol approved by the Institutional Animal Care and Use Committee of Kangwon National University (KIACUC, KW-130613-1).

## Author Contributions

H-JK, D-KS, and J-YJ designed the study. H-JL, H-JK, and J-YJ performed the experiments. H-JL and J-YJ wrote the paper with input from the other authors.

## Conflict of Interest Statement

The authors declare that the research was conducted in the absence of any commercial or financial relationships that could be construed as a potential conflict of interest.

## References

[1] Getahun H Matteelli A Abubakar I Aziz MA Baddeley A Barreira D Management of latent *Mycobacterium tuberculosis* infection: WHO guidelines for low tuberculosis burden countries. Eur Respir J (2015) 46(6):1563–76. 10.1183/13993003.01245-2015 26405286 PMC4664608

[2] Zumla A George A Sharma V Herbert RH Baroness Masham of I Oxley A The WHO 2014 global tuberculosis report – further to go. Lancet Glob Health (2015) 3(1):e10–2. 10.1016/S2214-109X(14)70361-4 25539957

[3] Stanley SA Cox JS. Host-pathogen interactions during *Mycobacterium tuberculosis* infections. Curr Top Microbiol Immunol (2013) 374:211–41. 10.1007/82_2013_332 23881288

[4] Stamm CE Collins AC Shiloh MU. Sensing of *Mycobacterium tuberculosis* and consequences to both host and bacillus. Immunol Rev (2015) 264(1):204–19. 10.1111/imr.12263 25703561 PMC4339209

[5] Goldberg MF Saini NK Porcelli SA Molecular Genetics of Mycobacteria. 2nd ed. Washington, DC: American Society Microbiology Press (2014).

[6] Trimble WS Grinstein S. TB or not TB: calcium regulation in mycobacterial survival. Cell (2007) 130(1):12–4. 10.1016/j.cell.2007.06.039 17632049

[7] Nunes P Demaurex N. The role of calcium signaling in phagocytosis. J Leukoc Biol (2010) 88(1):57–68. 10.1189/jlb.0110028 20400677

[8] Dussurget O Stewart G Neyrolles O Pescher P Young D Marchal G. Role of *Mycobacterium tuberculosis* copper-zinc superoxide dismutase. Infect Immun (2001) 69(1):529–33. 10.1128/IAI.69.1.529-533.2001 11119546 PMC97912

[9] Arandjelovic S Ravichandran KS. Phagocytosis of apoptotic cells in homeostasis. Nat Immunol (2015) 16(9):907–17. 10.1038/ni.3253 26287597 PMC4826466

[10] Lu N Zhou Z. Membrane trafficking and phagosome maturation during the clearance of apoptotic cells. Int Rev Cell Mol Biol (2012) 293:269–309. 10.1016/B978-0-12-394304-0.00013-0 22251564 PMC3551535

[11] Thi EP Reiner NE. Phosphatidylinositol 3-kinases and their roles in phagosome maturation. J Leukoc Biol (2012) 92(3):553–66. 10.1189/jlb.0212053 22569897

[12] Jaconi ME Lew DP Carpentier JL Magnusson KE Sjogren M Stendahl O. Cytosolic free calcium elevation mediates the phagosome-lysosome fusion during phagocytosis in human neutrophils. J Cell Biol (1990) 110(5):1555–64. 10.1083/jcb.110.5.1555 2110568 PMC2200167

[13] Malik ZA Denning GM Kusner DJ. Inhibition of Ca(2+) signaling by *Mycobacterium tuberculosis* is associated with reduced phagosome-lysosome fusion and increased survival within human macrophages. J Exp Med (2000) 191(2):287–302. 10.1084/jem.191.2.287 10637273 PMC2195750

[14] Kabarowski JH. G2A and LPC: regulatory functions in immunity. Prostaglandins Other Lipid Mediat (2009) 89(3–4):73–81. 10.1016/j.prostaglandins.2009.04.007 19383550 PMC2740801

[15] Radu CG Yang LV Riedinger M Au M Witte ON. T cell chemotaxis to lysophosphatidylcholine through the G2A receptor. Proc Natl Acad Sci U S A (2004) 101(1):245–50. 10.1073/pnas.2536801100 14681556 PMC314170

[16] Yang LV Radu CG Wang L Riedinger M Witte ON. Gi-independent macrophage chemotaxis to lysophosphatidylcholine via the immunoregulatory GPCR G2A. Blood (2005) 105(3):1127–34. 10.1182/blood-2004-05-1916 15383458

[17] Frasch SC Zemski-Berry K Murphy RC Borregaard N Henson PM Bratton DL. Lysophospholipids of different classes mobilize neutrophil secretory vesicles and induce redundant signaling through G2A. J Immunol (2007) 178(10):6540–8. 10.4049/jimmunol.178.10.6540 17475884

[18] Peter C Waibel M Radu CG Yang LV Witte ON Schulze-Osthoff K Migration to apoptotic “find-me” signals is mediated via the phagocyte receptor G2A. J Biol Chem (2008) 283(9):5296–305. 10.1074/jbc.M706586200 18089568

[19] Kabarowski JHS Zhu K Le LQ Witte ON Xu Y Lysophosphatidylcholine as a ligand for the immunoregulatory receptor G2A. *Science* (2001) 293(5530):702–5. 10.1126/science.1061781. 11474113

[20] Yan JJ Jung JS Lee JE Lee J Huh SO Kim HS Therapeutic effects of lysophosphatidylcholine in experimental sepsis. Nat Med (2004) 10(2):161–7. 10.1038/nm989 14716308

[21] Miyazaki H Midorikawa N Fujimoto S Miyoshi N Yoshida H Matsumoto T. Antimicrobial effects of lysophosphatidylcholine on methicillin-resistant *Staphylococcus aureus*. Ther Adv Infect Dis (2017) 4(4):89–94. 10.1177/2049936117714920 28748087 PMC5507393

[22] Hong CW Kim TK Ham HY Nam JS Kim YH Zheng H Lysophosphatidylcholine increases neutrophil bactericidal activity by enhancement of azurophil granule-phagosome fusion via glycine.GlyR alpha 2/TRPM2/p38 MAPK signaling. J Immunol (2010) 184(8):4401–13. 10.4049/jimmunol.0902814 20237295

[23] Tounsi N Meghari S Moser M Djerdjouri B. Lysophosphatidylcholine exacerbates *Leishmania major*-dendritic cell infection through interleukin-10 and a burst in arginase1 and indoleamine 2,3-dioxygenase activities. Int Immunopharmacol (2015) 25(1):1–9. 10.1016/j.intimp.2015.01.006 25601495

[24] Brancucci NMB Gerdt JP Wang C De Niz M Philip N Adapa SR Lysophosphatidylcholine regulates sexual stage differentiation in the human malaria parasite *Plasmodium falciparum*. Cell (2017) 171(7):1532–1544.e15. 10.1016/j.cell.2017.10.020 29129376 PMC5733390

[25] National Research Council. Guide for the Care and Use of Laboratory Animals. Washington, DC: National Academies Press (2011).

[26] Lee HJ Ko HJ Jung YJ. Insufficient generation of mycobactericidal mediators and inadequate level of phagosomal maturation are related with susceptibility to virulent *Mycobacterium tuberculosis* infection in mouse macrophages. Front Microbiol (2016) 7:541. 10.3389/fmicb.2016.00541 27148227 PMC4834433

[27] Lee HJ Kim KC Han JA Choi SS Jung YJ. The early induction of suppressor of cytokine signaling 1 and the downregulation of toll-like receptors 7 and 9 induce tolerance in costimulated macrophages. Mol Cells (2015) 38(1):26–32. 10.14348/molcells.2015.2136 25518931 PMC4314129

[28] Hong CW Song DK Immunomodulatory actions of lysophosphatidylcholine. Biomol Ther (2008) 16(2):69–76. 10.4062/biomolther.2008.16.2.069

[29] North RJ Jung YJ. Immunity to tuberculosis. Annu Rev Immunol (2004) 22:599–623. 10.1146/annurev.immunol.22.012703.104635 15032590

[30] Blander JM Medzhitov R. Regulation of phagosome maturation by signals from toll-like receptors. Science (2004) 304(5673):1014–8. 10.1126/science.1096158 15143282

[31] Vasiljeva O Reinheckel T Peters C Turk D Turk V Turk B. Emerging roles of cysteine cathepsins in disease and their potential as drug targets. Curr Pharm Des (2007) 13(4):387–403. 10.2174/138161207780162962 17311556

[32] Greenwell-Wild T Vazquez N Sim D Schito M Chatterjee D Orenstein JM *Mycobacterium avium* infection and modulation of human macrophage gene expression. J Immunol (2002) 169(11):6286–97. 10.4049/jimmunol.169.11.6286 12444135

[33] Santic M Molmeret M Kwaik YA. Maturation of the *Legionella pneumophila*-containing phagosome into a phagolysosome within gamma interferon-activated macrophages. Infect Immun (2005) 73(5):3166–71. 10.1128/Iai.73.5.3166-3171.2005 15845527 PMC1087382

[34] Benes P Vetvicka V Fusek M Cathepsin D – many functions of one aspartic protease. Crit Rev Oncol Hematol (2008) 68(1):12–28. 10.1016/j.critrevonc.2008.02.008 18396408 PMC2635020

[35] Stossel TP. Quantitative studies of phagocytosis. Kinetic effects of cations and heat-labile opsonin. J Cell Biol (1973) 58(2):346–56. 10.1083/jcb.58.2.346 4738105 PMC2109045

[36] Serezani CH Ballinger MN Aronoff DM Peters-Golden M. Cyclic AMP: master regulator of innate immune cell function. Am J Respir Cell Mol Biol (2008) 39(2):127–32. 10.1165/rcmb.2008-0091TR 18323530 PMC2720142

[37] Raker VK Becker C Steinbrink K. The cAMP pathway as therapeutic target in autoimmune and inflammatory diseases. Front Immunol (2016) 7:123. 10.3389/fimmu.2016.00123 27065076 PMC4814577

[38] Wall EA Zavzavadjian JR Chang MS Randhawa B Zhu XC Hsueh RC Suppression of LPS-induced TNF-alpha production in macrophages by cAMP is mediated by PKA-AKAP95-p105. Sci Signal (2009) 2(75):ra28. 10.1126/scisignal.2000202 19531803 PMC2770900

[39] Shabb JB Physiological substrates of cAMP-dependent protein kinase. Chem Rev (2001) 101(8):2381–411. 10.1021/cr000236l 11749379

[40] Meyer zu Heringdorf D Jakobs KH. Lysophospholipid receptors: signalling, pharmacology and regulation by lysophospholipid metabolism. Biochim Biophys Acta (2007) 1768(4):923–40. 10.1016/j.bbamem.2006.09.026 17078925

[41] Zhang P Katz J Michalek SM. Glycogen synthase kinase-3beta (GSK3beta) inhibition suppresses the inflammatory response to *Francisella* infection and protects against tularemia in mice. Mol Immunol (2009) 46(4):677–87. 10.1016/j.molimm.2008.08.281 18929413 PMC3033759

[42] Bai GC Schaak DD McDonough KA. cAMP levels within *Mycobacterium tuberculosis* and *Mycobacterium bovis* BCG increase upon infection of macrophages. FEMS Immunol Med Microbiol (2009) 55(1):68–73. 10.1111/j.1574-695X.2008.00500.x 19076221 PMC3222459

[43] Feng S Du YQ Zhang L Zhang L Feng RR Liu SY. Analysis of serum metabolic profile by ultra-performance liquid chromatography-mass spectrometry for biomarkers discovery: application in a pilot study to discriminate patients with tuberculosis. Chin Med J (Engl) (2015) 128(2):159–68. 10.4103/0366-6999.149188 25591556 PMC4837832

[44] Wood PL Tippireddy S Feriante J Plasma lipidomics of tuberculosis patients: altered phosphatidylcholine remodeling. Future Sci OA (2018) 4(1):FSO255 10.4155/fsoa-2017-0011 29255627 PMC5729594

[45] Parra Millan R Jimenez Mejias ME Sanchez Encinales V Ayerbe Algaba R Gutierrez Valencia A Pachon Ibanez ME Efficacy of lysophosphatidylcholine in combination with antimicrobial agents against *Acinetobacter baumannii* in experimental murine peritoneal sepsis and pneumonia models. Antimicrob Agents Chemother (2016) 60(8):4464–70. 10.1128/AAC.02708-15 27161639 PMC4958192

[46] Kalamidas SA Kuehnel MP Peyron P Rybin V Rauch S Kotoulas OB cAMP synthesis and degradation by phagosomes regulate actin assembly and fusion events: consequences for mycobacteria. J Cell Sci (2006) 119(Pt 17):3686–94. 10.1242/jcs.03091 16931599

[47] Vergne I Chua J Deretic V. Tuberculosis toxin blocking phagosome maturation inhibits a novel Ca^2+^/calmodulin-PI3K hVPS34 cascade. J Exp Med (2003) 198(4):653–9. 10.1084/jem.20030527 12925680 PMC2194170

[48] Vergne I Fratti RA Hill PJ Chua J Belisle J Deretic V. *Mycobacterium tuberculosis* phagosome maturation arrest: mycobacterial phosphatidylinositol analog phosphatidylinositol mannoside stimulates early endosomal fusion. Mol Biol Cell (2004) 15(2):751–60. 10.1091/mbc.E03-05-0307 14617817 PMC329390

[49] Fratti RA Chua J Deretic V. Induction of p38 mitogen-activated protein kinase reduces early endosome autoantigen 1 (EEA1) recruitment to phagosomal membranes. J Biol Chem (2003) 278(47):46961–7. 10.1074/jbc.M305225200 12963735

[50] Cavalli V Vilbois F Corti M Marcote MJ Tamura K Karin M The stress-induced MAP kinase p38 regulates endocytic trafficking via the GDI:Rab5 complex. Mol Cell (2001) 7(2):421–32. 10.1016/S1097-2765(01)00189-7 11239470

[51] Tejle K Magnusson KE Rasmusson B. Phagocytosis and phagosome maturation are regulated by calcium in J774 macrophages interacting with unopsonized prey. Biosci Rep (2002) 22(5–6):529–40. 10.1023/A:1022025903688 12635850

[52] Jope RS Yuskaitis CJ Beurel E. Glycogen synthase kinase-3 (GSK3): inflammation, diseases, and therapeutics. Neurochem Res (2007) 32(4–5):577–95. 10.1007/s11064-006-9128-5 16944320 PMC1970866

[53] Guha M Mackman N. The phosphatidylinositol 3-kinase-Akt pathway limits lipopolysaccharide activation of signaling pathways and expression of inflammatory mediators in human monocytic cells. J Biol Chem (2002) 277(35):32124–32. 10.1074/jbc.M203298200 12052830

[54] Martin M Rehani K Jope RS Michalek SM. Toll-like receptor-mediated cytokine production is differentially regulated by glycogen synthase kinase 3. Nat Immunol (2005) 6(8):777–84. 10.1038/ni1221 16007092 PMC1933525

[55] Chan MM Cheung BK Li JC Chan LL Lau AS. A role for glycogen synthase kinase-3 in antagonizing mycobacterial immune evasion by negatively regulating IL-10 induction. J Leukoc Biol (2009) 86(2):283–91. 10.1189/jlb.0708442 19401395

[56] Aliberti J Bafica A. Anti-inflammatory pathways as a host evasion mechanism for pathogens. Prostaglandins Leukot Essent Fatty Acids (2005) 73(3–4):283–8. 10.1016/j.plefa.2005.05.018 15982863

[57] Cooper AM Mayer-Barber KD Sher A. Role of innate cytokines in mycobacterial infection. Mucosal Immunol (2011) 4(3):252–60. 10.1038/mi.2011.13 21430655 PMC3294290

[58] Jung YJ Ryan L LaCourse R North RJ. Increased interleukin-10 expression is not responsible for failure of T helper 1 immunity to resolve airborne *Mycobacterium tuberculosis* infection in mice. Immunology (2003) 109(2):295–9. 10.1046/j.1365-2567.2003.01645.x 12757625 PMC1782960

[59] Redford PS Boonstra A Read S Pitt J Graham C Stavropoulos E Enhanced protection to *Mycobacterium tuberculosis* infection in IL-10-deficient mice is accompanied by early and enhanced Th1 responses in the lung. Eur J Immunol (2010) 40(8):2200–10. 10.1002/eji.201040433 20518032 PMC3378704

