# Supplementary material for: Lysophosphatidylcholine Promotes Phagosome Maturation and Regulates Inflammatory Mediator Production Through the Protein Kinase A–Phosphatidylinositol 3 Kinase–p38 Mitogen-Activated Protein Kinase Signaling Pathway During Mycobacterium tuberculosis Infection in Mouse Macrophages
Source: Front Immunol. 2018 Apr 27;9:920. doi: 10.3389/fimmu.2018.00920 (PMC5934435; doi:10.3389/fimmu.2018.00920)
Supplement: Supplementary file 1 [file image_1.PDF]

Supplementary Material

Lysophosphatidylcholine (LPC) promotes phagosome maturation and regulates inflammation through the PKA-PI3K-p38 MAPK signaling pathway during *Mycobacterium tuberculosis* infection in mouse macrophages

Hyo-Ji Lee[1,2], Hyun-Jeong Ko[3], Dong-Kun Song[4] and Yu-Jin Jung[1*]

*** Correspondence:**

Corresponding Author :

Yu-Jin Jung

yjjung@kangwon.ac.kr

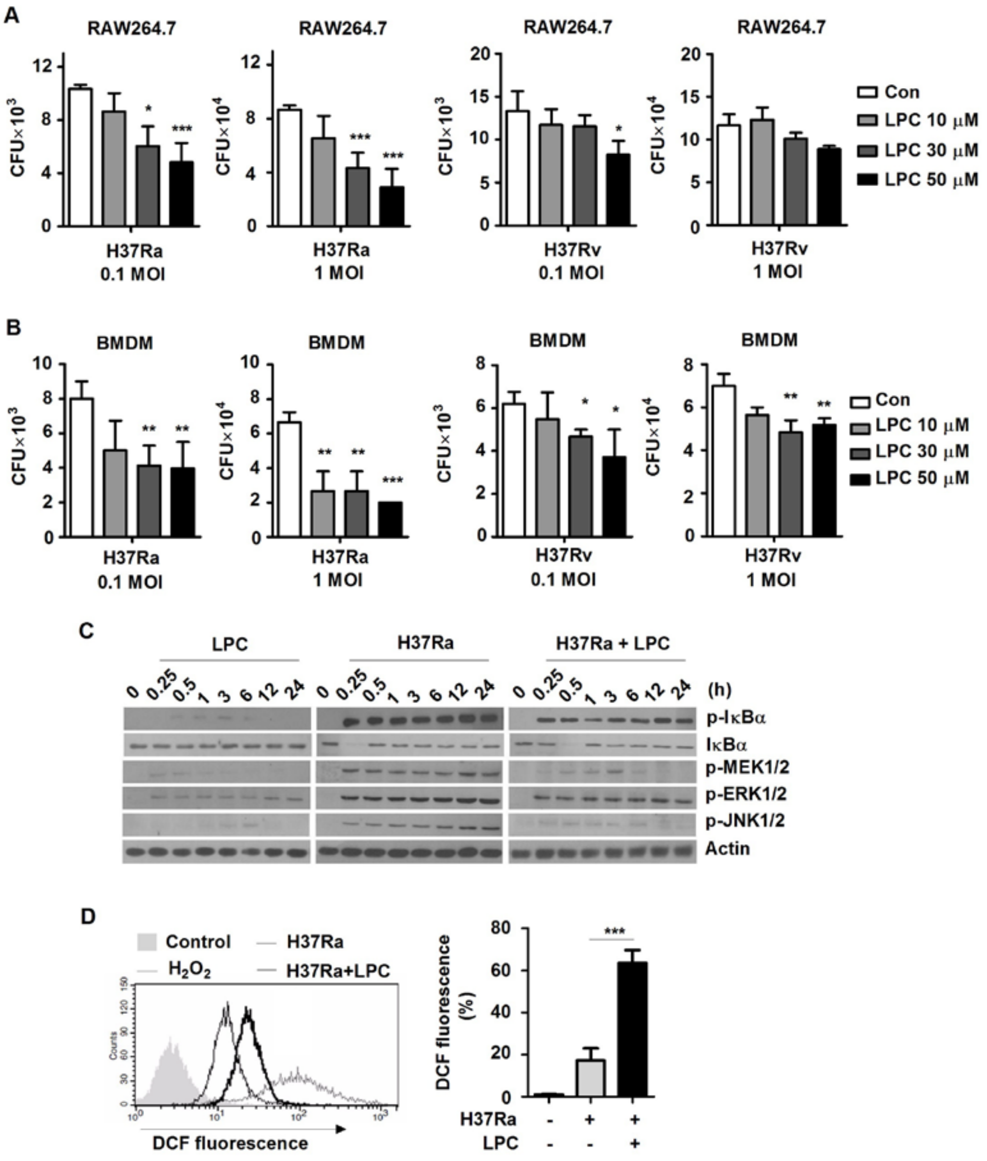

Supplementary Figure 1. LPC regulates intracellular bacterial growth and ROS production in Mtb-infected mouse macrophages. Raw264.7 cells (A) and BMDMs (B) isolated from C57BL/6 were stimulated with different doses of LPC for 24 h after infection with avirulent Mtb H37Ra or virulent Mtb H37Rv (at MOIs of 0.1 and 1). (A, B) Intracellular bacteria were assayed for viability based on the number of CFUs after 3 weeks. (C) Phosphorylated and total protein levels of the various components of the NF-κB and MAPK signaling pathways were examined by Western blot analysis in LPC-treated Raw264.7 cells during H37Ra infection. (D) The histogram overlay shows intracellular ROS in macrophages infected with H37Ra followed by treatment with LPC for 30 min. Cells were labeled with DCFH-DA and then measured by flow cytometry. The bar graph represents the percentage of DCF fluorescence. *, p<0.05; **, p<0.01; and ***, p<0.001.
