# Supplementary material for: Lysophosphatidylcholine Promotes Phagosome Maturation and Regulates Inflammatory Mediator Production Through the Protein Kinase A–Phosphatidylinositol 3 Kinase–p38 Mitogen-Activated Protein Kinase Signaling Pathway During Mycobacterium tuberculosis Infection in Mouse Macrophages
Source: Front Immunol. 2018 Apr 27;9:920. doi: 10.3389/fimmu.2018.00920 (PMC5934435; doi:10.3389/fimmu.2018.00920)
Supplement: Supplementary file 2 [file image_2.PDF]

Supplementary Material

Lysophosphatidylcholine (LPC) promotes phagosome maturation and regulates inflammation through the PKA-PI3K-p38 MAPK signaling pathway during *Mycobacterium tuberculosis* infection in mouse macrophages

Hyo-Ji Lee[1,2], Hyun-Jeong Ko[3], Dong-Kun Song[4] and Yu-Jin Jung[1*]

*** Correspondence:**

Corresponding Author :

Yu-Jin Jung

yjjung@kangwon.ac.kr

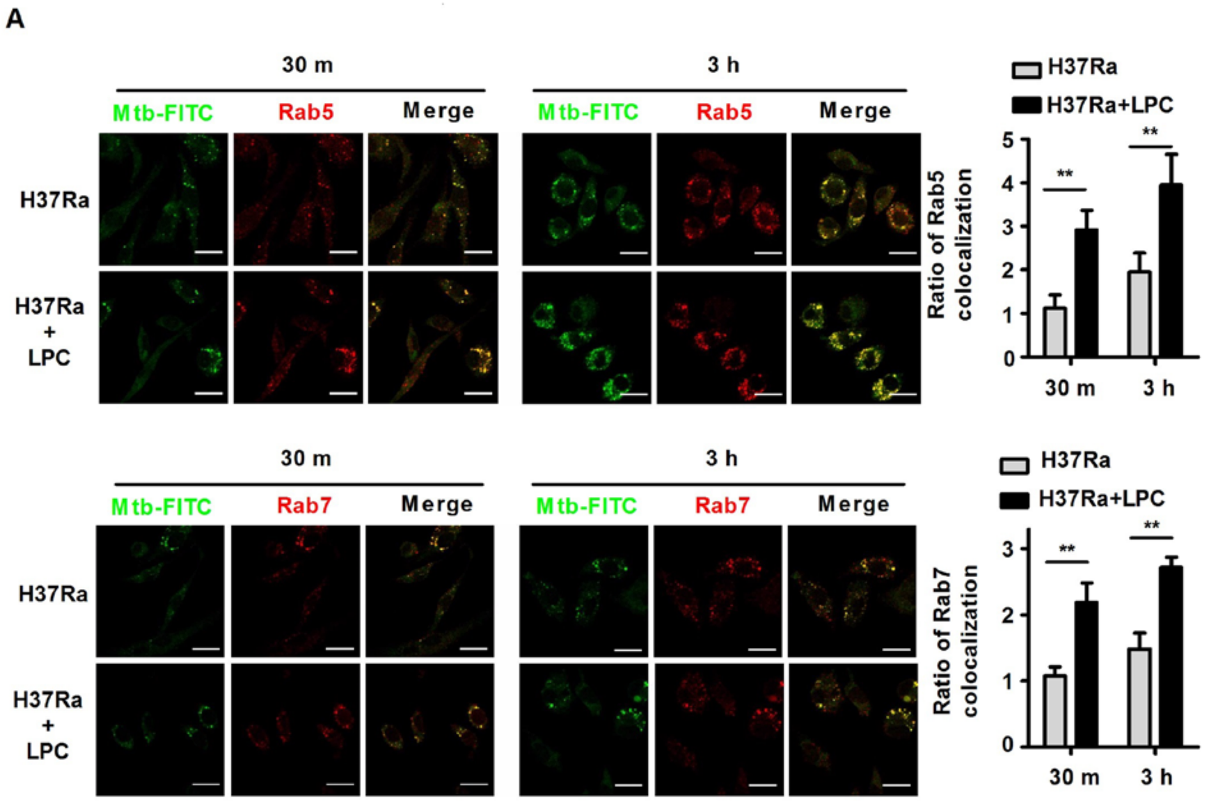

Supplementary Figure 2. LPC promotes phagosome maturation in Mtb-infected Raw264.7 cells. (A) Raw264.7 cells were infected with FITC-labeled H37Ra (MOI of 5) and treated with LPC for 30 min or 3 h. After infection, the cells were stained with another early phagosomal marker, Rab5 (top panel), and a late phagosomal marker, Rab7 (bottom panel), and Mtb colocalization with each marker was then observed by confocal microscopy. The bar graphs represent the ratio of Mtb colocalization with each marker. **, p<0.01.
