# Supplementary material for: Lysophosphatidylcholine Promotes Phagosome Maturation and Regulates Inflammatory Mediator Production Through the Protein Kinase A–Phosphatidylinositol 3 Kinase–p38 Mitogen-Activated Protein Kinase Signaling Pathway During Mycobacterium tuberculosis Infection in Mouse Macrophages
Source: Front Immunol. 2018 Apr 27;9:920. doi: 10.3389/fimmu.2018.00920 (PMC5934435; doi:10.3389/fimmu.2018.00920)
Supplement: Supplementary file 3 [file image_3.PDF]

Supplementary Material

Lysophosphatidylcholine (LPC) promotes phagosome maturation and regulates inflammation through the PKA-PI3K-p38 MAPK signaling pathway during *Mycobacterium tuberculosis* infection in mouse macrophages

Hyo-Ji Lee[1,2], Hyun-Jeong Ko[3], Dong-Kun Song[4] and Yu-Jin Jung[1*]

*** Correspondence:**

Corresponding Author :

Yu-Jin Jung

yjjung@kangwon.ac.kr

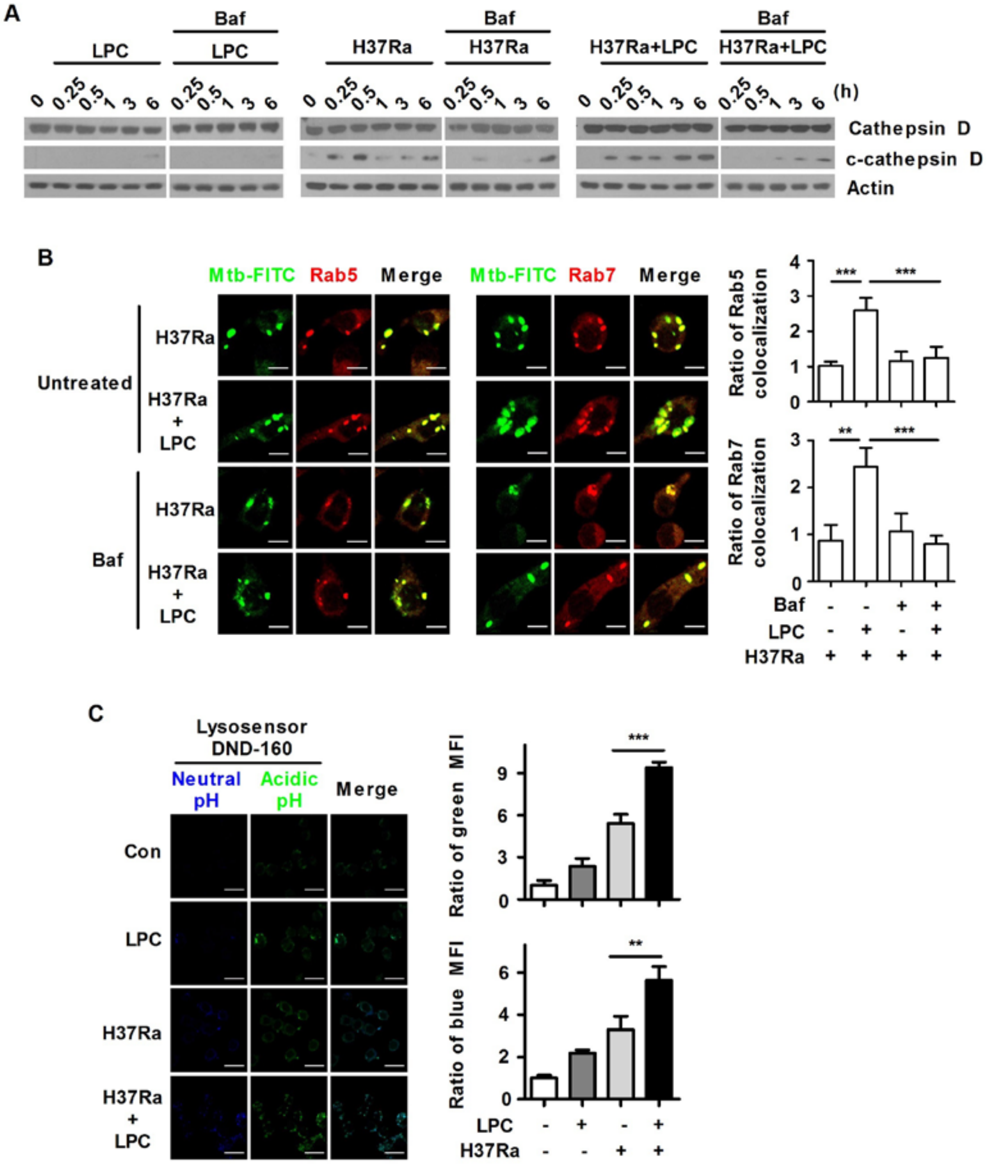

Supplementary Figure 3. LPC enhances phagosomal acidification in H37Ra-infected Raw264.7 cells. (A) After pre-treatment with bafilomycin A1 (Baf, 1 μM for 2 h), Raw264.7 cells were infected with H37Ra and treated with LPC for the indicated times. The expression level of cleaved cathepsin D (c-cathepsin D) was detected by Western blot analysis. (B) Raw264.7 cells were infected with FITC-labeled H37Ra and treated with LPC for 3 h. The cells were then stained with Rab5 or Rab7, and the accumulation of each phagosome marker was observed by confocal microscopy. The bar graphs represent the ratio of Mtb colocalization with each phagosome maturation marker. (C) The level of phagosomal acidification was detected using LysoSensor blue/yellow DND-160, a dual-emission wavelength dye. The bar graphs represent the ratio of green (acidic pH) or blue (neutral pH) mean fluorescence intensity (MFI), which was normalized to the MFI obtained for the uninfected cells. **, p<0.01 and ***, p<0.001.
