# Supplementary material for: Lysophosphatidylcholine Promotes Phagosome Maturation and Regulates Inflammatory Mediator Production Through the Protein Kinase A–Phosphatidylinositol 3 Kinase–p38 Mitogen-Activated Protein Kinase Signaling Pathway During Mycobacterium tuberculosis Infection in Mouse Macrophages
Source: Front Immunol. 2018 Apr 27;9:920. doi: 10.3389/fimmu.2018.00920 (PMC5934435; doi:10.3389/fimmu.2018.00920)
Supplement: Supplementary file 4 [file image_4.PDF]

Supplementary Material

Lysophosphatidylcholine (LPC) promotes phagosome maturation and regulates inflammation through the PKA-PI3K-p38 MAPK signaling pathway during *Mycobacterium tuberculosis* infection in mouse macrophages

Hyo-Ji Lee[1,2], Hyun-Jeong Ko[3], Dong-Kun Song[4] and Yu-Jin Jung[1*]

*** Correspondence:**

Corresponding Author :

Yu-Jin Jung

yjjung@kangwon.ac.kr

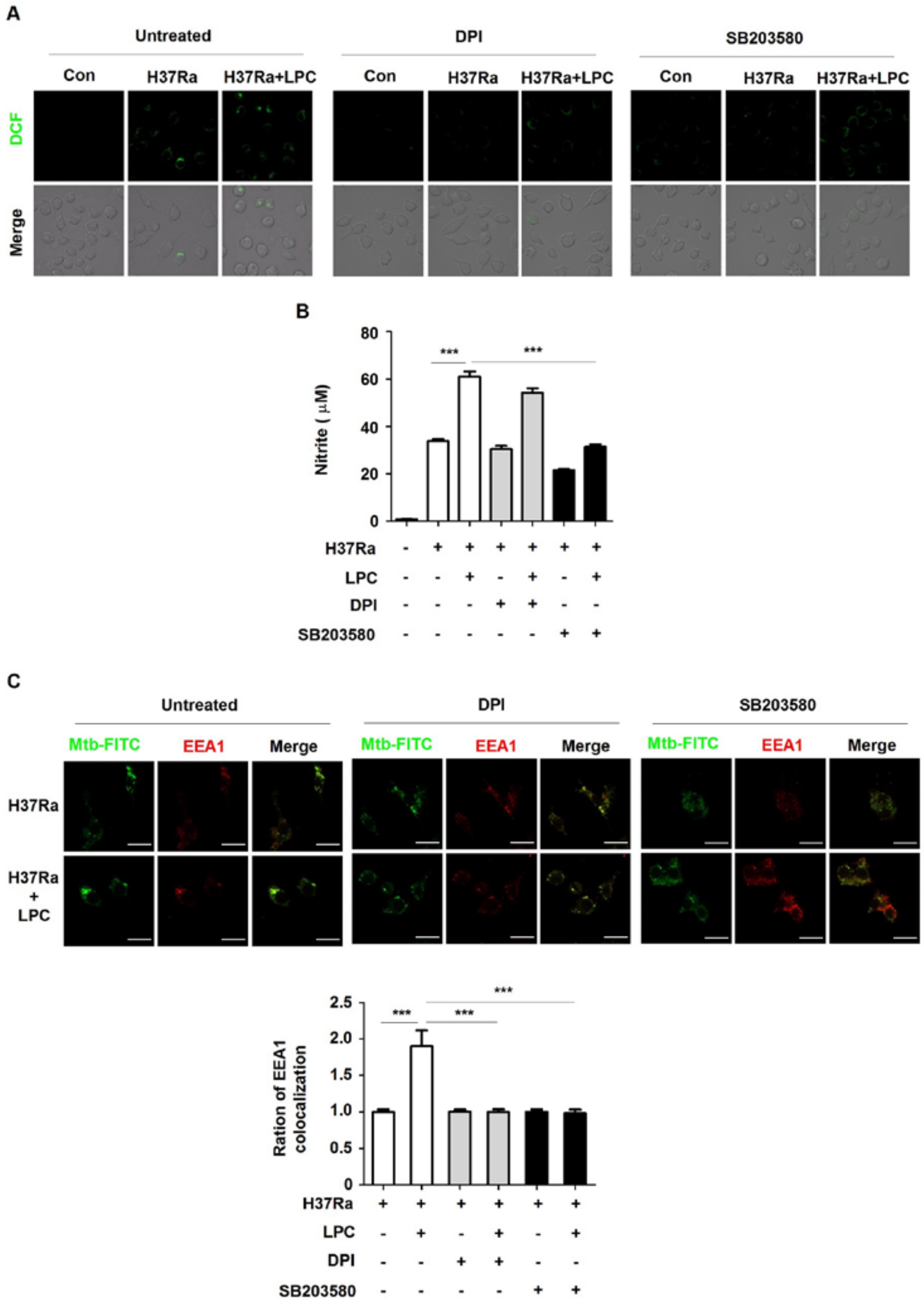

Supplementary Figure 4. LPC promotes phagosome maturation through PI3K-p38 MAPK-induced ROS production in H37Ra-infected macrophages. (A) Raw264.7 cells were pre-treated with SB203580 (10 μM) or DPI (10 μM) for 1 h and then stimulated with LPC during H37Ra infection (MOI of 5). After infection, the cells were labeled with DCFH-DA and then measured by confocal microscopy. (B) NO production was detected in LPC-treated Raw264.7 cells in the presence or absence of each inhibitor at 24 h post-infection. (C) After infection, the cells were stained with EEA1, and all images were viewed by confocal microscopy. The bar graph represents the ratio of Mtb colocalization with EEA1. ***, $p<0.001$.
