# Supplementary material for: Lysophosphatidylcholine Promotes Phagosome Maturation and Regulates Inflammatory Mediator Production Through the Protein Kinase A–Phosphatidylinositol 3 Kinase–p38 Mitogen-Activated Protein Kinase Signaling Pathway During Mycobacterium tuberculosis Infection in Mouse Macrophages
Source: Front Immunol. 2018 Apr 27;9:920. doi: 10.3389/fimmu.2018.00920 (PMC5934435; doi:10.3389/fimmu.2018.00920)
Supplement: Supplementary file 5 [file image_5.PDF]

Supplementary Material

Lysophosphatidylcholine (LPC) promotes phagosome maturation and regulates inflammation through the PKA-PI3K-p38 MAPK signaling pathway during *Mycobacterium tuberculosis* infection in mouse macrophages

Hyo-Ji Lee[1,2], Hyun-Jeong Ko[3], Dong-Kun Song[4] and Yu-Jin Jung[1*]

*** Correspondence:**

Corresponding Author :

Yu-Jin Jung

yjjung@kangwon.ac.kr

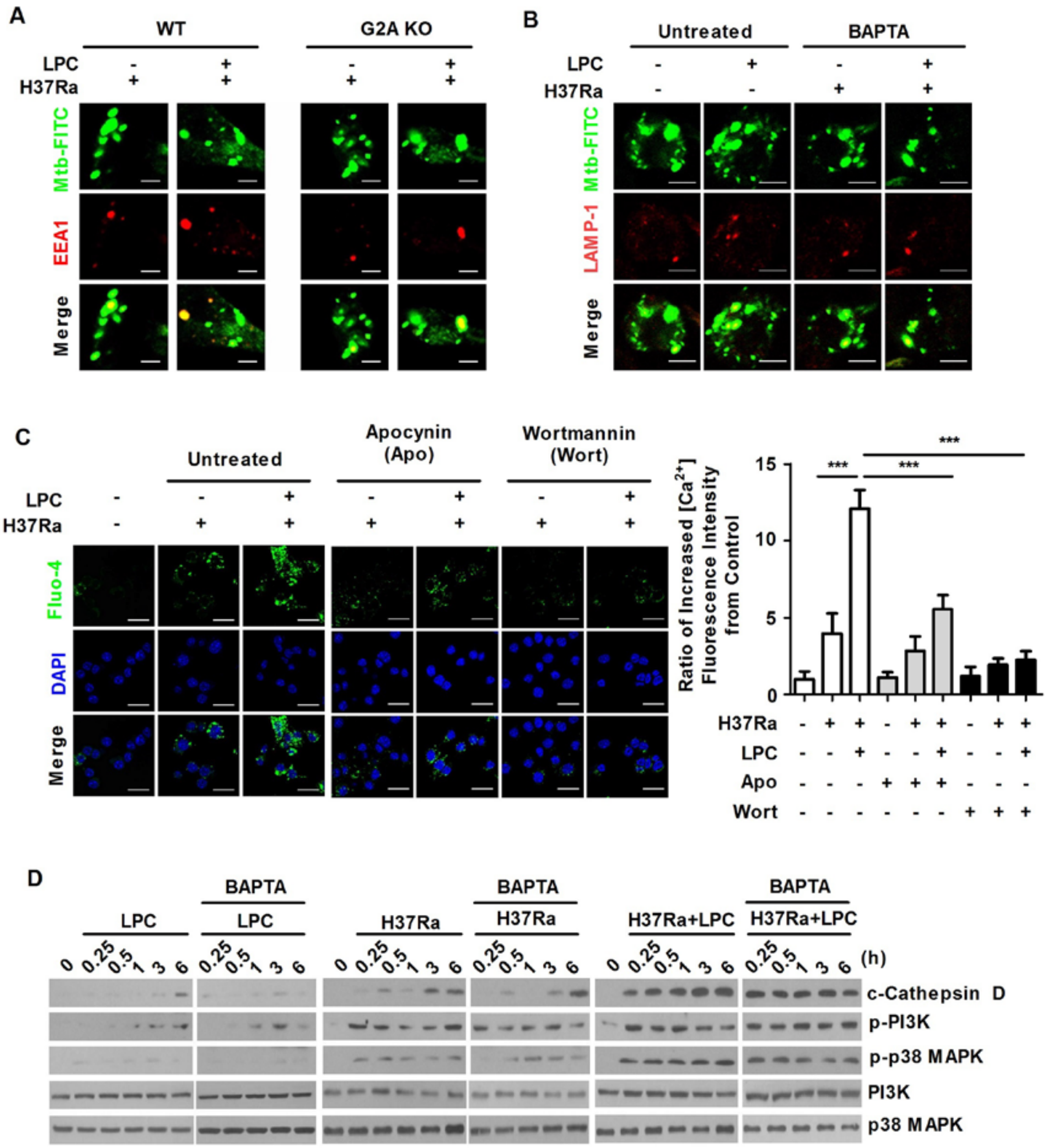

Supplementary Figure 5. Intracellular Ca^{2+} is involved in enhanced phagosome maturation via PI3K-p38 MAPK in LPC-treated macrophages during H37Ra infection. (A) WT and G2A KO BMDMs were infected with FITC-labeled H37Ra with or without LPC treatment for 3 h. After infection, the cells were stained with EEA1, and all images were viewed by confocal microscopy. (B) Raw264.7 cells were pre-treated with BAPTA/AM (30 μM) for 30 min and stimulated with LPC during FITC-labeled H37Ra infection (MOI of 5). After infection, the cells were stained with LAMP-1, and all images were observed by confocal microscopy. (C) Raw264.7 cells were pre-treated with wortmannin (Wort) or apocynin (Apo) and treated with LPC for 30 min during H37Ra infection (MOI of 5). After infection, the cells were loaded with Fluo-4/AM and stained with the nuclear dye DAPI. The bar graph represents the ratio of the Fluo-4/AM mean fluorescence intensity (MFI), which was normalized to the MFI obtained for uninfected cells. (D) Whole-cell lysates were analyzed by Western blot analysis of the indicated proteins. ***, $p<0.001$.
