# Supplementary material for: Lysophosphatidylcholine Promotes Phagosome Maturation and Regulates Inflammatory Mediator Production Through the Protein Kinase A–Phosphatidylinositol 3 Kinase–p38 Mitogen-Activated Protein Kinase Signaling Pathway During Mycobacterium tuberculosis Infection in Mouse Macrophages
Source: Front Immunol. 2018 Apr 27;9:920. doi: 10.3389/fimmu.2018.00920 (PMC5934435; doi:10.3389/fimmu.2018.00920)
Supplement: Supplementary file 6 [file image_6.PDF]

Supplementary Material

Lysophosphatidylcholine (LPC) promotes phagosome maturation and regulates inflammation through the PKA-PI3K-p38 MAPK signaling pathway during *Mycobacterium tuberculosis* infection in mouse macrophages

Hyo-Ji Lee[1,2], Hyun-Jeong Ko[3], Dong-Kun Song[4] and Yu-Jin Jung[1*]

*** Correspondence:**

Corresponding Author :

Yu-Jin Jung

yjjung@kangwon.ac.kr

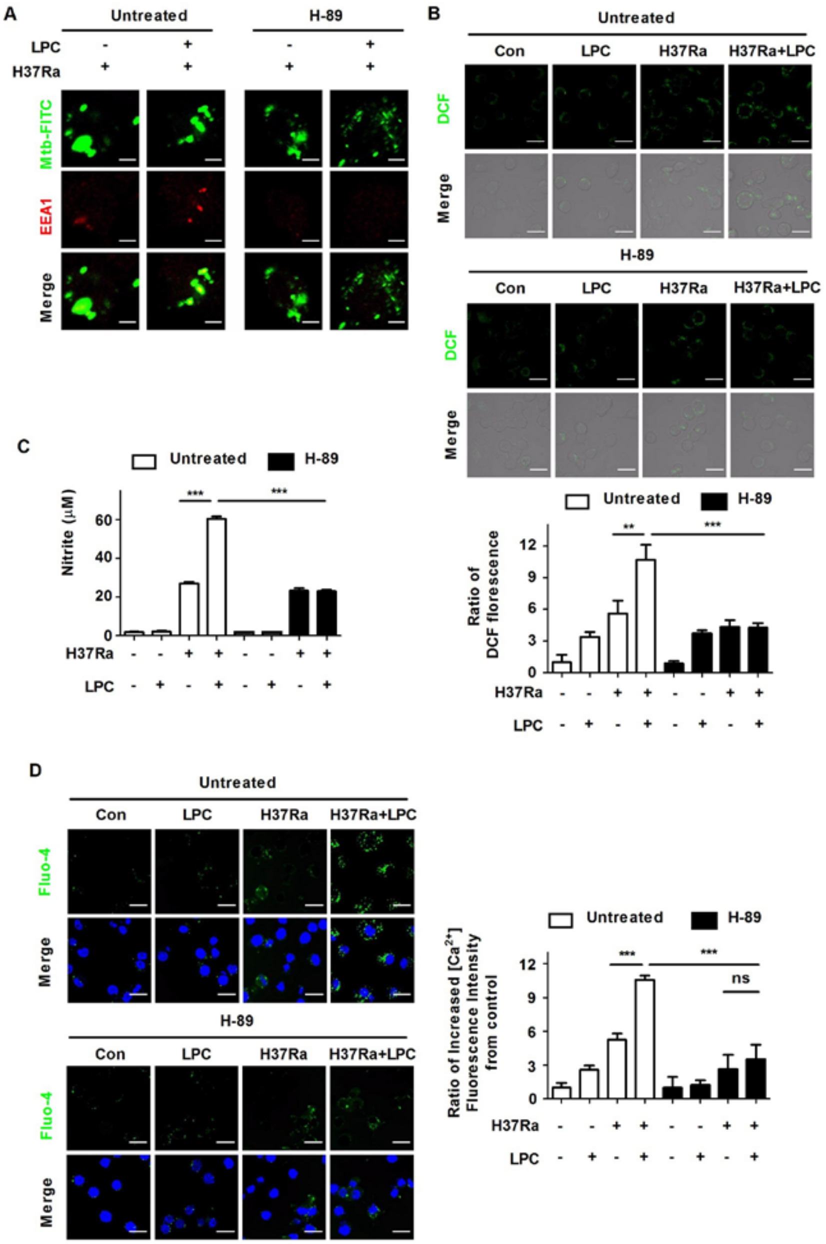

Supplementary Figure 6. LPC regulates the levels of ROS and NO and intracellular Ca^{2+} release through cAMP-induced activation of PKA in Mtb-infected cells. Raw264.7 cells were pre-treated with H-89 (10 μM) for 1 h and infected with H37Ra (MOI of 5) with or without LPC treatment. (A) After infection, the cells were stained with EEA1, and FITC-labeled Mtb colocalization with EEA1 was observed by confocal microscopy. (B) The cells were labeled with DCFH-DA, and intracellular ROS levels were measured by confocal microscopy. The bar graph represents the ratio of DCF fluorescence. (C) NO was measured in cell culture supernatants using the NO detection kit. (D) Cells were loaded with Fluo-4/AM and stained with the nuclear dye DAPI. Intracellular Ca^{2+} release was observed by confocal microscopy. The bar graph represents the ratio of the Fluo-4/AM MFI, which was normalized to the MFI obtained for uninfected cells. **, *p*<0.01; ***, *p*<0.001; and ns, not significant (*p*>0.05).
