# Supplementary material for: Lysophosphatidylcholine Promotes Phagosome Maturation and Regulates Inflammatory Mediator Production Through the Protein Kinase A–Phosphatidylinositol 3 Kinase–p38 Mitogen-Activated Protein Kinase Signaling Pathway During Mycobacterium tuberculosis Infection in Mouse Macrophages
Source: Front Immunol. 2018 Apr 27;9:920. doi: 10.3389/fimmu.2018.00920 (PMC5934435; doi:10.3389/fimmu.2018.00920)
Supplement: Supplementary file 7 [file image_7.PDF]

Supplementary Material

Lysophosphatidylcholine (LPC) promotes phagosome maturation and regulates inflammation through the PKA-PI3K-p38 MAPK signaling pathway during *Mycobacterium tuberculosis* infection in mouse macrophages

Hyo-Ji Lee[1,2], Hyun-Jeong Ko[3], Dong-Kun Song[4] and Yu-Jin Jung[1*]

*** Correspondence:**

Corresponding Author :

Yu-Jin Jung

yjjung@kangwon.ac.kr

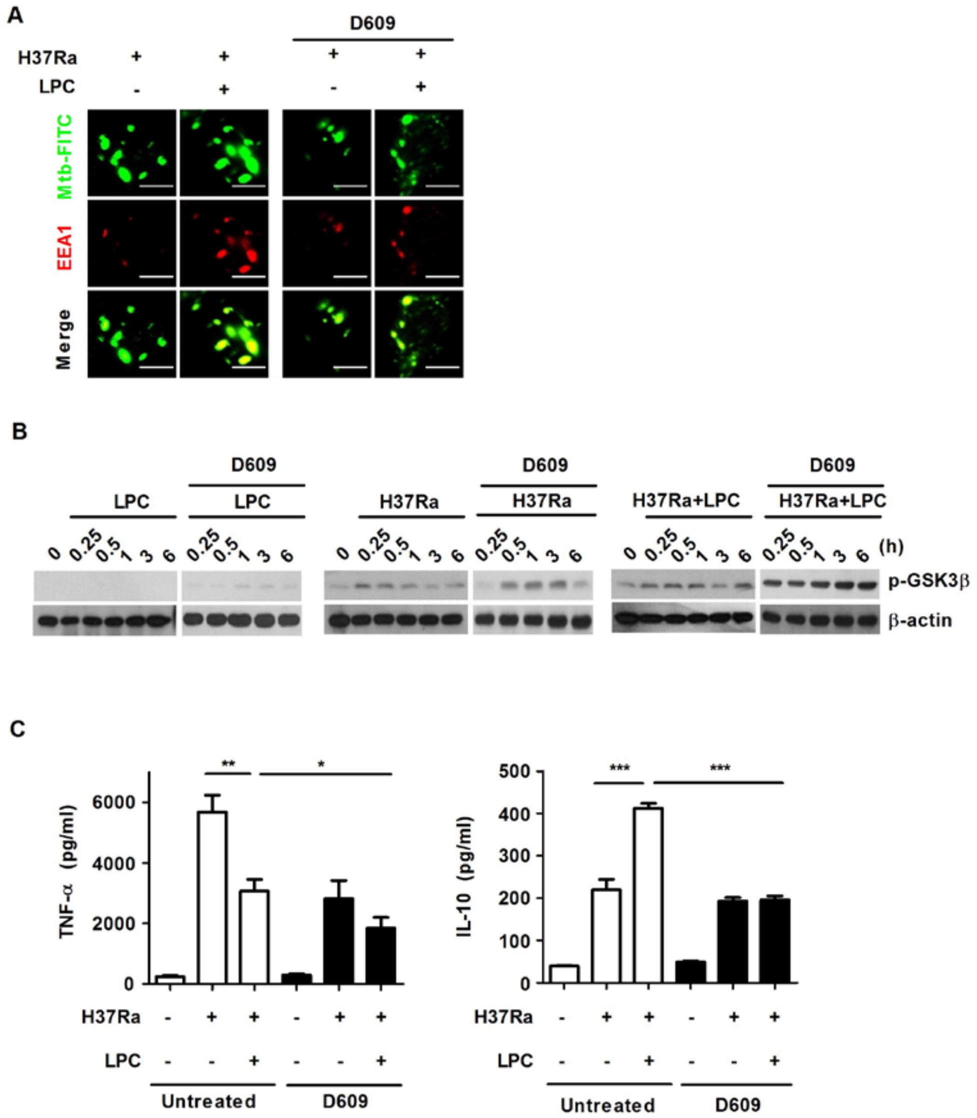

Supplementary Figure 7. LPC regulates the production of inflammatory mediators via G2A-mediated activation of PLC during H37Ra infection. Raw264.7 cells were pre-treated with D609 for 12 h and stimulated with LPC during H37Ra infection (MOI of 5). (A) After infection, the cells were stained with EEA1, and FITC-labeled Mtb colocalization with EEA1 was observed by confocal microscopy. (B) Whole-cell lysates were analyzed by Western blot analysis of the indicated proteins. (C) The production of TNF-α and IL-10 was measured in the cell culture medium. *, $p<0.05$; **, $p<0.01$; and ***, $p<0.001$.
