# Supplementary material for: Lysophosphatidylcholine Promotes Phagosome Maturation and Regulates Inflammatory Mediator Production Through the Protein Kinase A–Phosphatidylinositol 3 Kinase–p38 Mitogen-Activated Protein Kinase Signaling Pathway During Mycobacterium tuberculosis Infection in Mouse Macrophages
Source: Front Immunol. 2018 Apr 27;9:920. doi: 10.3389/fimmu.2018.00920 (PMC5934435; doi:10.3389/fimmu.2018.00920)
Supplement: Supplementary file 8 [file image_8.PDF]

Supplementary Material

Lysophosphatidylcholine (LPC) promotes phagosome maturation and regulates inflammation through the PKA-PI3K-p38 MAPK signaling pathway during *Mycobacterium tuberculosis* infection in mouse macrophages

Hyo-Ji Lee[1,2], Hyun-Jeong Ko[3], Dong-Kun Song[4] and Yu-Jin Jung[1*]

*** Correspondence:**

Corresponding Author :

Yu-Jin Jung

yjjung@kangwon.ac.kr

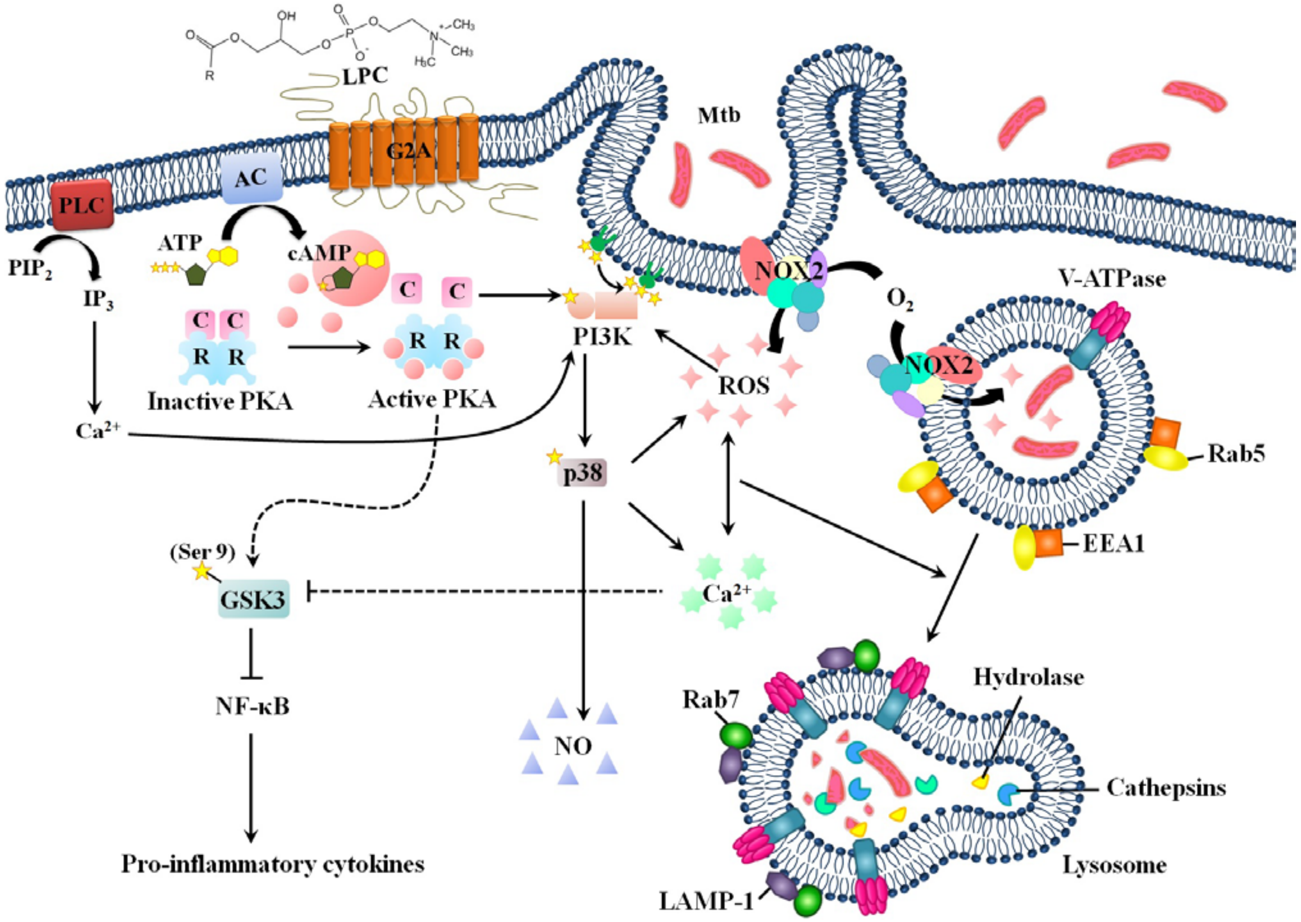

Supplementary Figure 8. Schematic model proposing the role of LPC in regulating phagosome maturation and inflammatory mediators in Mtb-infected macrophages. The model depicts that LPC induces an increased cAMP production, which is responsible for activation of the PI3K-p38 MAPK signaling pathway during Mtb infection. LPC also accelerates phagosome maturation by up-regulating ROS and cytosolic Ca^{2+} release through PLC activity and PKA-PI3K-p38 MAPK signaling during Mtb H37Ra infection. Taken together, LPC controls Mtb growth without an excessive production of inflammatory mediators by enhancing the phosphorylation of GSK3β through PLC activity and the PKA-PI3K-p38 MAPK signaling pathway during Mtb infection.
